# Neuronal mechanisms for sequential activation of memory items: Dynamics and reliability

**DOI:** 10.1371/journal.pone.0231165

**Published:** 2020-04-16

**Authors:** Elif Köksal Ersöz, Carlos Aguilar, Pascal Chossat, Martin Krupa, Frédéric Lavigne

**Affiliations:** 1 Project Team MathNeuro, INRIA-CNRS-UNS, Sophia Antipolis, France; 2 Lab by MANTU, Amaris Research Unit, Route des Colles, Biot, France; 3 Université Côte d’Azur, Laboratoire Jean-Alexandre Dieudonné, Nice, France; 4 Université Côte d’Azur, CNRS-BCL, Nice, France; Sorbonne Universite UFR de Biologie, FRANCE

## Abstract

In this article we present a biologically inspired model of activation of memory items in a sequence. Our model produces two types of sequences, corresponding to two different types of cerebral functions: activation of regular or irregular sequences. The switch between the two types of activation occurs through the modulation of biological parameters, without altering the connectivity matrix. Some of the parameters included in our model are neuronal gain, strength of inhibition, synaptic depression and noise. We investigate how these parameters enable the existence of sequences and influence the type of sequences observed. In particular we show that synaptic depression and noise drive the transitions from one memory item to the next and neuronal gain controls the switching between regular and irregular (random) activation.

## Introduction

The processing of sequences of items in memory is a fundamental issue for the brain to generate sequences of stimuli necessary for goal-directed behavior [1], language processing [2, 3], musical performance [4, 5], thinking and decision making [6] and more generally prediction [7–9]. Those processes rely on priming mechanisms in which a triggering stimulus (e.g. a prime word) activates items in memory corresponding to stimuli not actually presented (e.g. target words) [10, 11]. A given triggering stimulus can generate two types of sequences: on the one hand, the systematic activation of the same sequence is required to repeat reliable behaviors [12–16]; on the other hand, the generation of variable sequences is necessary for the creation of new behaviors [17–21]. Hence the brain has to face two opposite constraints of generating repetitive sequences or of generating new sequences. Satisfying both constraints challenges the link between the types of sequence generated by the brain and the relevant biological parameters. Can a neural network with a fixed synaptic matrix switch behavior between reproducing a sequence and produce new sequences? And which neuronal mechanisms are sufficient for such switch in the type of sequence generated? The question addressed here is how changes in neuronal noise, short-term synaptic depression and neuronal gain make possible either repetitive or variable sequences.

Neural correlates of sequence processing involve cerebral cortical areas from V1 [16, 22] and V4 [14] to prefrontal, associative, and motor areas [23, 24]. The neuronal mechanisms involve a distributed coding of information about items across a pattern of activity of neurons [25–29]. In priming studies, neuronal activity recorded after presentation of a prime image shifts from neurons active for that image to neurons active for another image not presented, hence beginning a sequence of neuronal patterns [30–33]. Those experiments report that a condition for the shift between neuronal patterns of activity is that stimuli have been previously learned as being associated. Considering that the synaptic matrix codes the relation between items in memory [34, 35], computational models of priming have shown that the activation of sequences of two populations of neurons rely on the efficacy of the synapses between neurons from these two populations [10, 36–39].

Turning to longer sequences, many of the models studied to date rely on the existence of steady patterns (equilibria) of saddle type, which allow for transitions from one memory item to the next [40–42]. Such models are well suited for reproducing systematically the same unidirectional sequence: as time evolves neuronal patterns are activated in a systematic order. These works show that the generation of directional sequences relies on the asymmetry of the relations between the populations of neurons that are activated successively. Regarding the order of populations n, n+1, n+2 in a sequence, the directionality of the sequence is obtained thanks to two properties of the synaptic matrix. First, the synaptic efficacy increases with the order of the populations, that is efficacy is weaker between populations one and two than between populations two and three [15, 40]. Second, the amount of overlap increases with the order of populations [42]. Indeed, individual neurons respond to several different stimuli [43–45] and two populations of neurons coding for two items can share some active neurons [46, 47]. Models have proposed a Hebbian learning mechanism that determines synaptic efficacy as a function of the overlap between the populations [48, 49]. In models the amount of overlap codes for the association between the populations and determines their order of activation in a sequence [11, 40, 42, 50]. These works identify sufficient properties of the synaptic matrix to generate systematic sequences. However such properties of the synaptic matrix may not be necessary and neuronal mechanisms may also be sufficient to generate sequences.

Neural network models have pointed to neuronal gain as a key parameter that determines the easiness of state transitions and the stability of internal representations [51]. Further, a cortical network model has shown that neuronal gain determines the amount of activation between populations of neurons associated through potentiated synapses [52]. The latter has shown that variable values of gain reproduce the variable magnitude of the activation of associates in memory (semantic priming) reported in schizophrenic participants compared to healthy participants [53–55]. However, these models considered states stability or the amount of activation but not the reliability nor the length of the sequences that can be activated. This points to a possible effect of neuronal gain but leaves open the possibility that it could play a role in the regularity or variability of the sequences that can be activated.

In this work we consider the case of fixed synaptic efficacy and fixed overlap to focus on sufficient neuronal mechanisms that underlie the type of sequence, reliable or variable. The present study mathematically analyses a new and more general type of sequences in which the states of the network do not need to pass near saddle points. The model is based on a more general mechanism of transition from one memory item to the next, with the saddle pattern replaced by a saddle-sink pair (see [56], for a prototype of this mechanism of transition). As time evolves the sink and saddle patterns become increasingly similar, so that even a small random perturbation can push the system past the saddle to the next memory item. In the model those new dynamics alleviate constraints on the synaptic matrix by allowing sequences that form spontaneously with the transitions obtained between populations related through fixed overlap, without theoretical or practical restriction on the length of the sequences. We show that, in addition to regular (predictable) sequences which follow the overlap between the populations, our system also supports sequences with random transitions between learned patterns. We investigate how changes in parameters with a clear biological meaning such as neuronal noise, short-term synaptic depression (or short-term depression (STD), for short) and neuronal gain can control the reliability of the sequences.

Our model is mainly deterministic, however small noise is needed to facilitate transitions from one state to the next. As in [40] we used small noise to activate regular transitions, and, unlike in other contexts, e.g. [42], we used small noise for random activations. In the context of large noise (stochastic systems), it is difficult to generate regular sequences if white noise is used. This is the main reason why we decided to take an almost deterministic approach. As our goal was to understand the possible effects of deterministic dynamics, we chose time independent white noise.

## Model

The focus of this paper is to present a mechanism of sequential activation of memory items in the absence of either increasing overlap, or increasing synaptic conductance, or any other feature forcing directionality of the sequences. We present this mechanism in the context of a simple system, however the idea is general and can be implemented in detailed models. We use the neural network model of the form

$$\begin{array}{cc} & \dot{x_{i}}=x_{i}\left(1-x_{i}\right)(-\mu x_{i}-I-\lambda \sum_{j=1}^{N}x_{j}+\sum_{j=1}^{N}J_{i,j}^{max}s_{j}x_{j})+\eta \end{array}$$ (1)

$$\begin{array}{cc} & \dot{s_{i}}=\frac{1-s_{i}}{\tau_{r}}-Ux_{i}s_{i}\;\left(i=1,\cdots ,N\right), \end{array}$$ (2)

as in [40], with the variables *x*_*i*_ ∈ [0, 1] representing normalised averaged firing rates of excitatory neuronal populations (units), and *s*_*i*_ ∈ [0, 1] controlling STD. The limiting firing rates *x*_*i*_ = 0 and *x*_*i*_ = 1 correspond respectively to the resting and excited states of unit *i*. Any set (*x*_1_, …, *x*_*N*_) with *x*_*i*_ = 0 or 1 (*i* = 1, …, *N*) defines a steady, or equilibrium, *pattern* for the network. In the classical paradigm the learning process results in the formation of stable patterns of the network. Retrieving memory occurs when a cue puts the network in a state which belongs to the basin of attraction of the learned pattern. Eq (1) is usually formulated using the activity variable *u*_*i*_ (average membrane potential) rather than *x*_*i*_, and *x*_*i*_ is related to *u*_*i*_ through a sigmoid transfer function. Our formulation in which the inverse of the sigmoid is replaced by a linear function with slope *μ*, was shown to be convenient for finding sequential retrievals of learned patterns, see [40].

The parameters in Eq (1) are *μ* (or its inverse *γ* = *μ*^−1^ which is the gain, supposed identical, of the units, or slope of the activation function of the neuron [57]), λ the strength of a non-selective inhibition (inhibitory feedback due to excitation of interneurons) and $J_{i,j}^{max}$ the maximum weight of the connexion from unit *j* to unit *i*. The parameter *I* can be understood as feedforward inhibition [58] or distance to the excitability threshold. This parameter was used by [11, 40, 50]. Note that *I* controls the stability of the completely inactive state (*x*_*i*_ = 0 for all *i*). In this work we set *I* to 0, which means that the inactive state is marginally stable (see Section “Marginal stability of the inactive state” in S1 Appendix). This is reminiscent of the up state [59], characterised by neurons being close to the firing threshold. Finally, *η* is a noise term which can be thought of as a fluctuation of the firing rate due to random presence or suppression of spikes. In our simulations we considered white noise with the additional constraint of pointing towards the interior of the interval [0, 1]. Other types of noise can be chosen, this does not affect the mechanisms which we have investigated.

STD reported in cortical synapses [60] rapidly decreases the efficacy of synapses that transmit the activity of the pre-synaptic neuron. This is modeled by Eq (2) where *τ*_*r*_ is the synaptic time constant of the synapse and *U* is the fraction of used synaptic resources. In order to be more explicit for the rest of the manuscript, we re-write Eq (2) as

$$\begin{array}{c} \tau_{r}\dot{s_{i}}=1-s_{i}-\rho x_{i}s_{i}\;\left(i=1,\cdots ,N\right), \end{array}$$ (3)

where *ρ* = *τ*_*r*_ *U*. Eq (3) immediately shows the respective roles played by *τ*_*r*_ and the synaptic product *ρ*. The synaptic time constant *τ*_*r*_ produces slow dynamics when *τ*_*r*_ ≫ 1, while *ρ* determines the value of the limiting state of the synaptic strength. More precisely, for an active unit *x*_*i*_ = 1 with initially maximal synaptic strength *s*_*i*_ = 1, *s*_*i*_ decays towards the value *S* = (1 + *ρ*)^−1^ by following

$$\begin{array}{c} s_{i}\left(t\right)=1-\frac{1-\exp(-\left(1+\rho \right)t/\tau_{r})}{1+\rho }, \end{array}$$

with the decay time constant (1 + *ρ*)/*τ*_*r*_ which depends on *τ*_*r*_. For an inactive unit *x*_*i*_ = 0, *s*_*i*_ recovers to *s*_*i*_ = 1 by following

$$\begin{array}{c} s_{i}\left(t\right)=1-\left(1-s_{i}\left(0\right)\right)\exp\left(-t/\tau_{r}\right), \end{array}$$

with the recovery time constant *τ*_*r*_ and *s*_*i*_(0) is the synaptic value at the beginning of the recovery process.

The main difference in the model between this paper and [40] is the form of the matrix of excitatory connections *J*^max^:

$$\begin{array}{c} J^{max}={\left[\begin{array}{ccccc} 1 & 1 & 0 & \ldots & 0\\ 1 & 2 & 1 & \ddots & \vdots \\ 0 & \ddots & \ddots & \ddots & 0\\ \vdots & \ddots & 1 & 2 & 1\\ 0 & \ldots & 0 & 1 & 1 \end{array}\right]}_{N\times N}. \end{array}$$ (4)

This matrix is derived by the application of the simplified Hebbian learning rule of [61] (details provided in [40]) using the collection of learned patterns

$$\begin{array}{cc} & \xi^{i}=\left(0,\cdots ,0,1,1,0,\cdots ,0\right)\;,\;i=1,\cdots ,P \end{array}$$ (5)

where the two excited units are *i* and *i* + 1. Conditions for the stability of these patterns in the absence of STD were derived in [40]. Note that the overlap between *ξ*^*i*^ and *ξ*^*i*+1^ is constant (one unit). By the application of the learning rule the coefficients of *J*^*max*^ are given by the formula:

$$\begin{array}{c} J_{i,j}^{max}=\sum_{k=1}^{P}\xi_{i}^{k}\xi_{j}^{k}. \end{array}$$ (6)

Consequently the matrix *J*^*max*^ is made up of identical (1 2 1) blocks along the diagonal, so that there is no increase in either overlap or the synaptic efficacy (weight) along any possible chain. We prove mathematically and verify by numerics that Eq (1) admits a chain of latching dynamics passing through the patterns *ξ*^*i*^, *i* = 1, …*n* − 2, either in forwards or in backwards direction depending on the activation, as well as shorter chains. The simplest way to switch dynamically from the learned pattern *ξ*^*i*^ to *ξ*^*i*+1^ is by having a mechanism such that unit *i* passes from excited to rest state, then unit *i* + 2 passes from rest to excited state. STD can clearly result in the inhibition of unit *i*. However in the framework of [40] it was not possible to obtain the spontaneous excitation of unit *i* + 2 with the connectivity matrix Eq (4), because it was required that the upper and lower diagonal coefficients of *J*^*max*^ be strictly increasing with the order *i*.

Connectionist models have shown the effects of fast synaptic depression on semantic memory [62] and on priming [11, 50]. Recall that fast synaptic depression is one of the aspects of short term synaptic plasticity [63]. For the sake of simplicity, we neglect the other aspects of short term synaptic plasticity, but their effects on sequential activation of memory items are likely to be significant [64], and we intend to investigate them in further work. Fast synaptic depression contributes to deactivation of neurons initially active in a pattern—because they activate less and less each other—in favor of the activation of neurons active in a different but overlapping pattern—because newly activated neurons can strongly activate their associates in a new pattern. The combination of neuronal noise and fast synaptic depression enables latching dynamics in any direction depending on the initial bias due to random noise. Indeed, when the parameters lie within a suitable range, the action of STD has the effect of creating a “dynamic equilibrium” with a small basin of attraction. This dynamic equilibrium could be *ξ*^*i*^, ${\hat{\xi }}^{i}$ (the pattern in which only unit *i* + 1 is excited) or an intermediate pattern for which the value of *x*_*i*_ is between 0 and 1. Subsequently the noise allows the system to eventually jump to *ξ*^*i*+1^, the process being repeated sequentially between all or part of the learned patterns. This noise-driven transition is what we call an *excitable connection* by reference to a similar phenomenon discussed in [56]. Chains of excitable connections can also be activated or terminated by noise. Last but not least we show that our system, depending on the value of the parameter *μ*, hence of the neuronal gain *γ* = 1/*μ*, will follow the sequence indicated by the overlap or execute a random sequence of activations. Changes in neuronal gain change the sensitivity of a neuron to its incoming activation [57, 65, 66], and are reported to impact contextual processing [67] to enhance the quality of neuronal representations [68] and to modulate activation between populations of neurons to reproduce priming experiments [52]. Here we show how changes in neuronal gain switches the network’s behavior between repetitive (reliable) sequences and variable (new) sequences.

We proceed to present the results in more detail, as follows. In Sec. Case study: a system with *N* = 8 excitable units we present simulations for the network with *N* = 8, which serves as an example of the more general construction. In Sec. Case study: a system with *N* = 8 excitable units we sketch the methods we use to search for or verify the existence of the chains. In Sec. Irregular chains and additional numerical results we discuss irregular chains of random activations versus regular chains defined by the overlap. Simulations were run using the Euler-Maruyama method with time steps of 0.01 ms.

## Results

### Case study: A system with *N* = 8 excitable units

**Fig 1:**
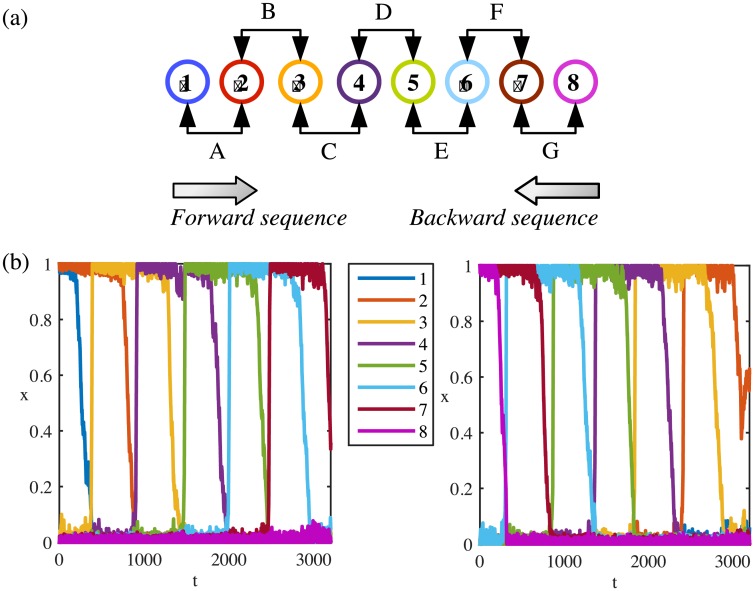
Directional sequences of an endpoint stimulus-driven system. (a) Each numbered circle represents a unit. Consecutive units encode a pattern. Except for *x*_1_ and *x*_8_, each unit participates in two patterns. A forward sequence is the activation of units in increasing order. A backward sequence is the activation of units in decreasing order. (b) Left panel: System initialised from the pattern A follows the forward sequence until the pattern F. Right panel: System initialised from the pattern G follows the backward sequence until the pattern B. Same colour code is used to represent units’ indices in (a) and (b). Parameters: *μ* = 0.41, λ = 0.51, *I* = 0, *ρ* = 1.8, *τ*_*r*_ = 900, *η* = 0.02.

We consider sequences of seven learned patterns *ξ*^1^, …, *ξ*^7^ (named ABCDEFG) encoded by eight units *x*_1_, …, *x*_8_. The sequence represents the sequential activation of pairs of units 1-2, 2-3, 3-4, 4-5, 5-6, 6-7 and 7-8, corresponding to patterns A and B, B and C, etc. with an overlap of one unit between them (see Fig 1a). Learning is reported to rely on changes in the efficacy of the synapses between neurons [69] through long term potentiation (LTP) and long term depression (LTD) [70–72]. As a consequence, LTP/LTD potentiates/depresses synapses between units coding for patterns as a function of their overlap, that is synapses between units coding for overlapping patterns are more potentiated. Due to the constant overlap, all synapses between overlapping patterns are equal. Note that the matrix *J*^*max*^ is learned as a function of the overlap between patterns without imposing any sequences. A consequence is that learning of independent pairs of patterns generates a matrix that allows for the activation of sequences.

A system of *N* = 8 excitatory units can encode *P* = *N* − 1 = 7 regular patterns in *J*^*max*^ (see Fig 1a). Encoded memory items can be retrieved either spontaneously (in a noisy environment) or when the memory network is triggered by an external cue [42, 73]. Units *x*_1_ and *x*_8_ are the least self-excited units with $J_{1,1}^{max}=J_{8,8}^{max}=1$, thus it is very unlikely to active them unless they are part of the initial activity state. Hence, the longest chain has *P* − 1 = 6 consecutive patterns.

#### Directional sequences from a stimulus-driven pattern in the sequence

Starting from the first pattern A, the directional activation corresponds to the sequence ABCDEFG (Fig 1b left panel). The forward direction is imposed by *J*^*max*^ because *x*_1_ is less excited since $J_{1,1}^{max}=1$. Hence, while the synaptic variables *s*_1_ and *s*_2_ are equal and decreasing together as the system lies in the vicinity of *ξ*^1^, *x*_1_ is deactivated before *x*_2_. In the same interval of time *s*_2_ < *s*_3_ and *s*_2_ − *s*_3_ increases so that *x*_2_ becomes unstable before *x*_3_ and the system now may converge to *ξ*^2^. The process can be repeated between *ξ*^2^ and *ξ*^3^ etc. Similarly, starting from the last pattern G gives the reverse direction (GFEDCBA) to the system (Fig 1b right panel).

**Fig 2:**
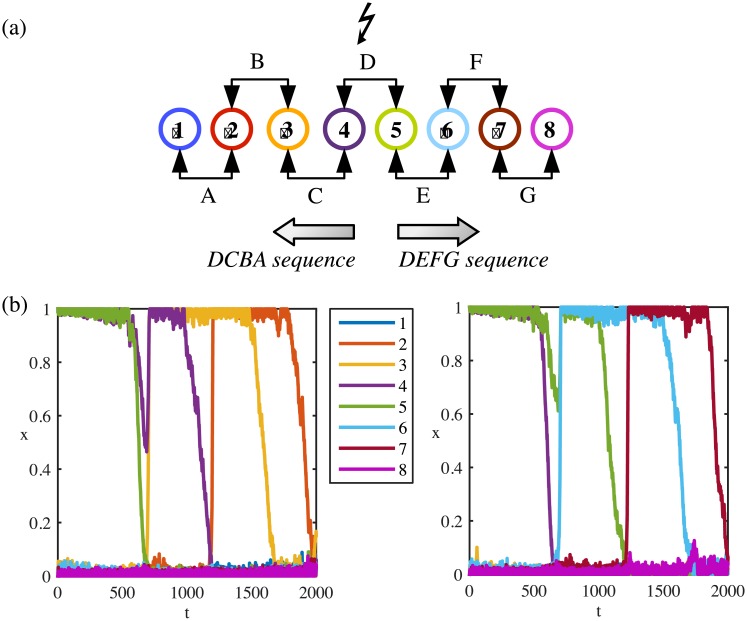
Directional sequences of a midpoint stimulus-driven system. (a) Each numbered circle represents a unit. Consecutive neurons encode a pattern. Except for *x*_1_ and *x*_8_, each unit participates in the encoding for two patterns. When the system initialised from the pattern D, it follows either the “DCBA” or “DEFG” sequence. (b) Left panel: System initialised from the pattern D follows the “DCBA” sequence until the pattern B. Right panel: System initialised from the pattern D follows “DEFG” sequence until the pattern F. The same colour code is used to represent units’ indices in (a) and (b). Parameters: *μ* = 0.414, λ = 0.51, *I* = 0, *ρ* = 1.8, *τ*_*r*_ = 900, *η* = 0.02.

Initialising the system from a middle pattern *ξ*^*i*^ does not introduce any direction, since the two active units of *ξ*^*i*^ are equally excited. While their synaptic variables are decreasing together, depending on the noise at the moment when *ξ*^*i*^ becomes unstable, either *ξ*^*i*−1^ or *ξ*^*i*+1^ is activated with equal probabilities. Fig 2 shows the response of the system starting from a mid-point pattern D. The activated sequence can go in either direction DEFG or its reverse DCBA. The *random* choice for a sequence is driven by a bias in the noise at the time of stimulus-driven activation of the mid-point pattern.

#### Noise-driven random sequence from a mid-point pattern in the sequence

**Fig 3:**
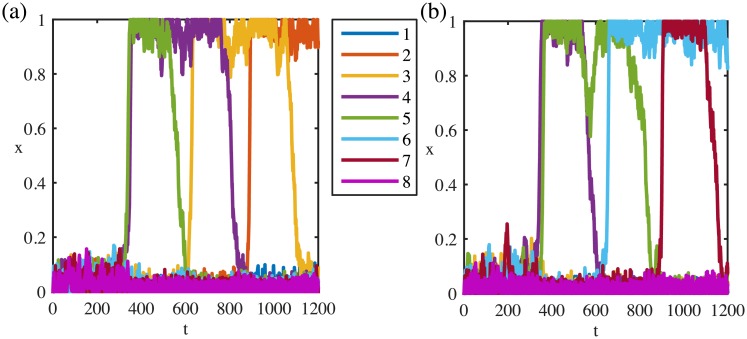
Directional sequences of a spontaneously activated system. System activated spontaneous by random noise can move in backward (a) or forward (b) directions. Parameters: *μ* = 0.21, λ = 0.51, *I* = 0, *ρ* = 1.8, *τ*_*r*_ = 300, *η* = 0.04.

The units that participate in two patterns (overlapping units) have stronger self-excitation as it is manifested by the diagonals of *J*^*max*^. These units (*x*_*i*_, *i* ≠ {1, 8}) are likely to be excited by random noise and they can activate others which they encode a pattern with. After a pattern *ξ*^*i*^ or the associated intermediate pattern ${\hat{\xi }}^{i}=\left(0,\ldots ,0,1,0,\ldots ,0\right)$ being randomly excited by noise, the system can follow either *ξ*^*i*−1^ or *ξ*^*i*+1^. The robustness of activity depends on the system parameters. Fig 3 shows an example of spontaneous activation of a mid-point pattern D where the directional oriented sequence can be either DEFG or its reverse DCBA. Similar to the system initialised from a middle pattern, the *random* choice for a direction is driven by a bias in the noise at the time of noise-driven activation.

#### Sensitivity of the dynamics upon parameter values

We have seen that patterns can be retrieved sequentially when the system is triggered by a cue or spontaneously by noise. However the effectiveness of this process depends on the values of the parameters in Eqs (1) and (2). The dynamics of the system can follow part of the sequence, then either terminate on one pattern *ξ*^*i*^ with *i* < *N* − 1, or converge to a non learned pattern. Moreover we identified two different dynamical scenarios by which a sequence can be followed, depending mainly on the value of *μ*. This will be analyzed in Sec. Analysis of the dynamics. Here we comment on numerical simulations which highlight the dependency of the sequences upon parameter values.

The behavior of the model was tested on simulation data by measuring the length of regular sequences generated by the network (chain length) and by computing the distance made from the initial pattern after irregular sequences (distance). Chain length and distance were analyzed by fitting linear mixed-effect models (LMM) to the data, using the lmer function from the lme4 package (Version 1.1–7) in R (Version R-3.1.3 [74]). All predictor parameters (inverse of the gain *μ*, inhibition λ, time constant *τ*_*r*_, synaptic constant *ρ* and noise *η*) were defined as continuous variables and they were centered on their mean. The optimal structure was determined after comparing the goodness of fit of a range of models, using Akaike’s information criterion (AIC); the model with the smallest AIC was selected, corresponding to the model with main effects and interactions between all of the parameters. The significance of the effects was tested using the lmerTest package. For the sake of clarity of the text, we flag the levels of significance with one star (*) if p-value <0.05, two stars (**) if p-value <0.01, three stars (***) if p-value<0.001.

**Fig 4:**
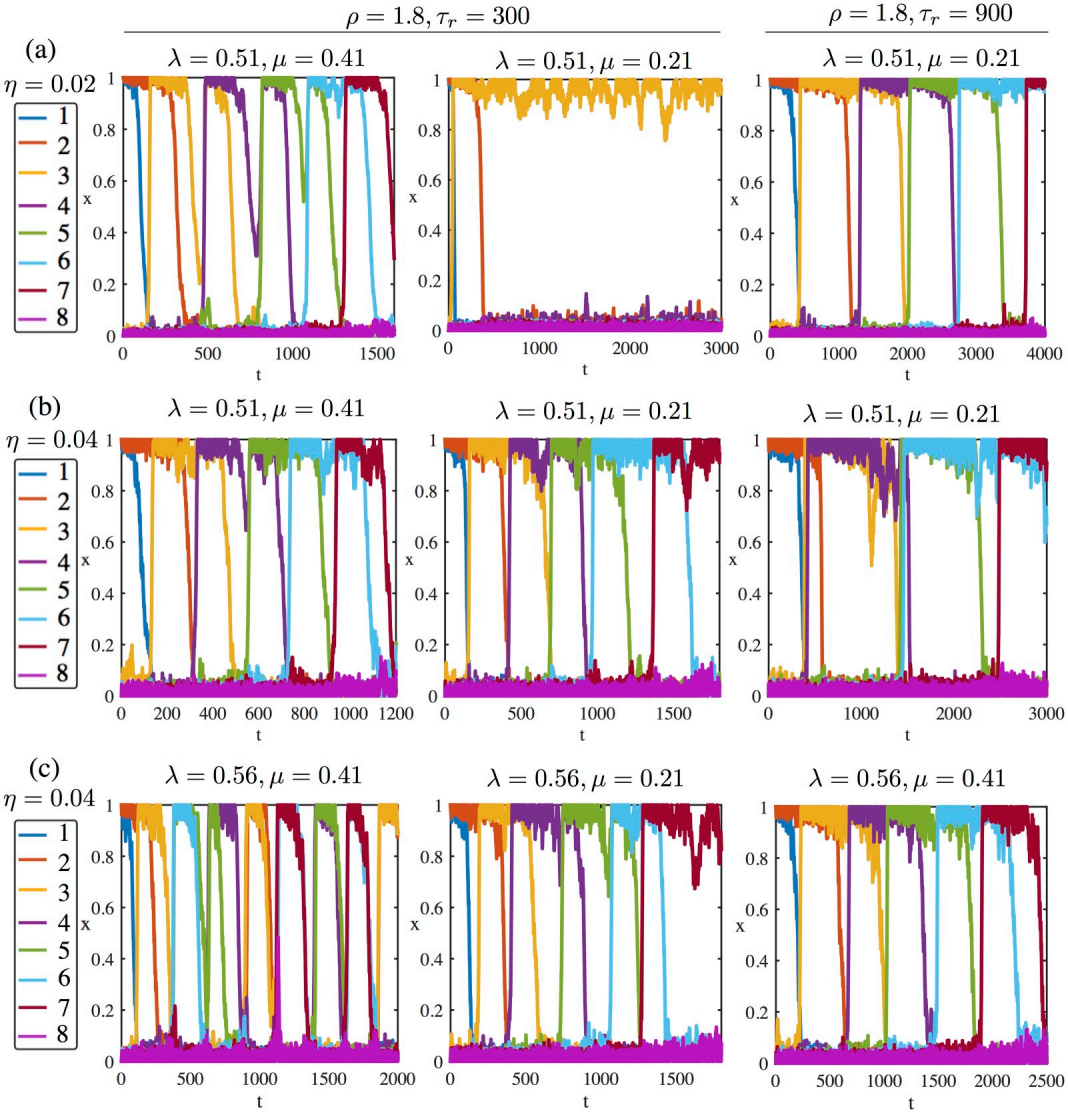
Response of the system initialised from pattern A (units 1-2) to different levels of noise *η* and system parameters λ, *μ*, *τ*_*r*_. Synaptic variables are faster along the first two columns (*τ*_*r*_ = 300) than the last column (*τ*_*r*_ = 900). In all simulations *I* = 0. Row (a) *η* = 0.02, λ = 0.51. The system with fast synapses can follow the longest sequence from A (units 1-2) to F (units 6-7) for *μ* = 0.41 (the first panel) but not for *μ* = 0.21 (the second panel), while the slow synapses can trigger the longest sequence (the third panel). Row (b) *η* = 0.04, λ = 0.51. Increasing the noise amplitude enables the activation of the whole sequence with fast synapses (the first two panels), whereas the slow synapses give either very short patterns or 3 co-active units, the third panel, respectively. Row (c) *η* = 0.04, λ = 0.56. Increasing the global inhibition λ regulates the transition for slow synapses (the third panel), whereas the system with fast synapses and *μ* = 0.41 (the first panel) randomly activates learned patterns and yields short regular and irregular sequences. On the other hand, the system with *μ* = 0.21 and fast synapses (the second panel) can preserve a regular sequence.

Fig 4 shows time series of the full or partial completion of sequences of retrievals (for *N* = 8 units) for two different values of noise amplitude *η* = 0.02 (first row) and *η* = 0.04 (second and third row). In each case the two first columns show time series with the STD parameters *ρ* = 1.8 and *τ*_*r*_ = 300 while the last column corresponds to the choice *ρ* = 1.8 and *τ*_*r*_ = 900. By fixing the synaptic constant *ρ* = 1.8, we ensure that the synaptic variables *s*_*i*_ decay to the same value *S* with a decay time depending on *τ*_*r*_ (see Sec. Model). The global inhibition coefficient λ is set at 0.51 in rows Fig 4a and 4b and λ = 0.56 in row Fig 4c. For each choice of the STD parameters *μ* takes two values, either *μ* = 0.41 or *μ* = 0.21.

Observe that the sequence and the pattern durations are shorter in the system with fast synapses (*τ*_*r*_ = 300) than the one with slow synapses (*τ*_*r*_ = 900). In the case of weaker noise (Fig 4a) and fast synapses, the system follows the sequence ABCDEF when *μ* = 0.41 whereas it stops at the pattern B when *μ* = 0.21. In other words, increasing *μ* (decreasing neural gain) in the system with fast synapses recruits more units sequentially. Another way to increase the chain length for *μ* = 0.21 is slowing down the synaptic variables. The system with slow synapses can follow the sequence ABCDEF for a wide range of *μ* values. In fact the two different values of *μ* in Fig 4 correspond to the two different dynamical scenarios which have been evoked in the beginning of this section. This point will be developed in Sec. Analysis of the dynamics. When noise is stronger (Fig 4b) the picture is different: the full sequence can be completed with fast synapses even for *μ* = 0.21. However, the sequence is shorter with slow synapses and the system quickly explores unexpected patterns like one with three excited units 2, 3, 4 around *t* = 600 (which is not a learned pattern) in third panel of Fig 4b. These type of activity can be observed for a wide range of *μ* values with slow synapses.

Comparison between Fig 4b and 4c exemplifies the effect of changing inhibition λ and *μ* (inverse of neural gain) for the same noise amplitude. Increasing the inhibition coefficient λ regulates the transition for slow synapses, while fast synapses and high values of *μ* (low neural gain) randomly activates the learned patterns and yields short sequences. The latter is also due to the self inhibition in the system given by the −*μx*_*i*_ term in Eq (1) which facilitates deactivation of an active unit, but makes difficult for an inactive unit to be activated if it is too high. Notice the self inhibitory effect in the fast synapses can be compensated for slow synapses and small *μ* (high neural gain) which can regularize sequences.

#### Length of a chain

When the patterns in a chain are explored in the right order by the system we call it *regular*. As we saw in Sec. Sensitivity of the dynamics upon parameter values it can happen that only part of the full regular chain has been realised before it stops or starts exploring patterns in a different order, hence activating an irregular chain. We call the partial regular chain a *regular segment* and its length is the number of patterns it contains. Here we investigate the maximal length that a regular segment starting at pattern A can attain. This length is the rank of the last activated pattern over simulations. It depends on noise *η* but also on the neuronal parameters (*μ*, λ) and on the synaptic parameters (*τ*_*r*_, *ρ*). As it can be read in Eq (3), *ρ* characterizes the limiting decay state of the synaptic variable. Possible impact of *τ*_*r*_ and *ρ* on the dynamics has been investigated in [40]. It has been shown in a system with a a structured connectivity matrix that deactivation of a unit is harder for *ρ* being small while too high *ρ* prevents recruiting new units. Thus, *ρ* determines the reliability of a sequential activation. On the other hand, the threshold of the noise need for activation of a chain decreases with increasing *τ*_*r*_ for *ρ* constant.

**Fig 5:**
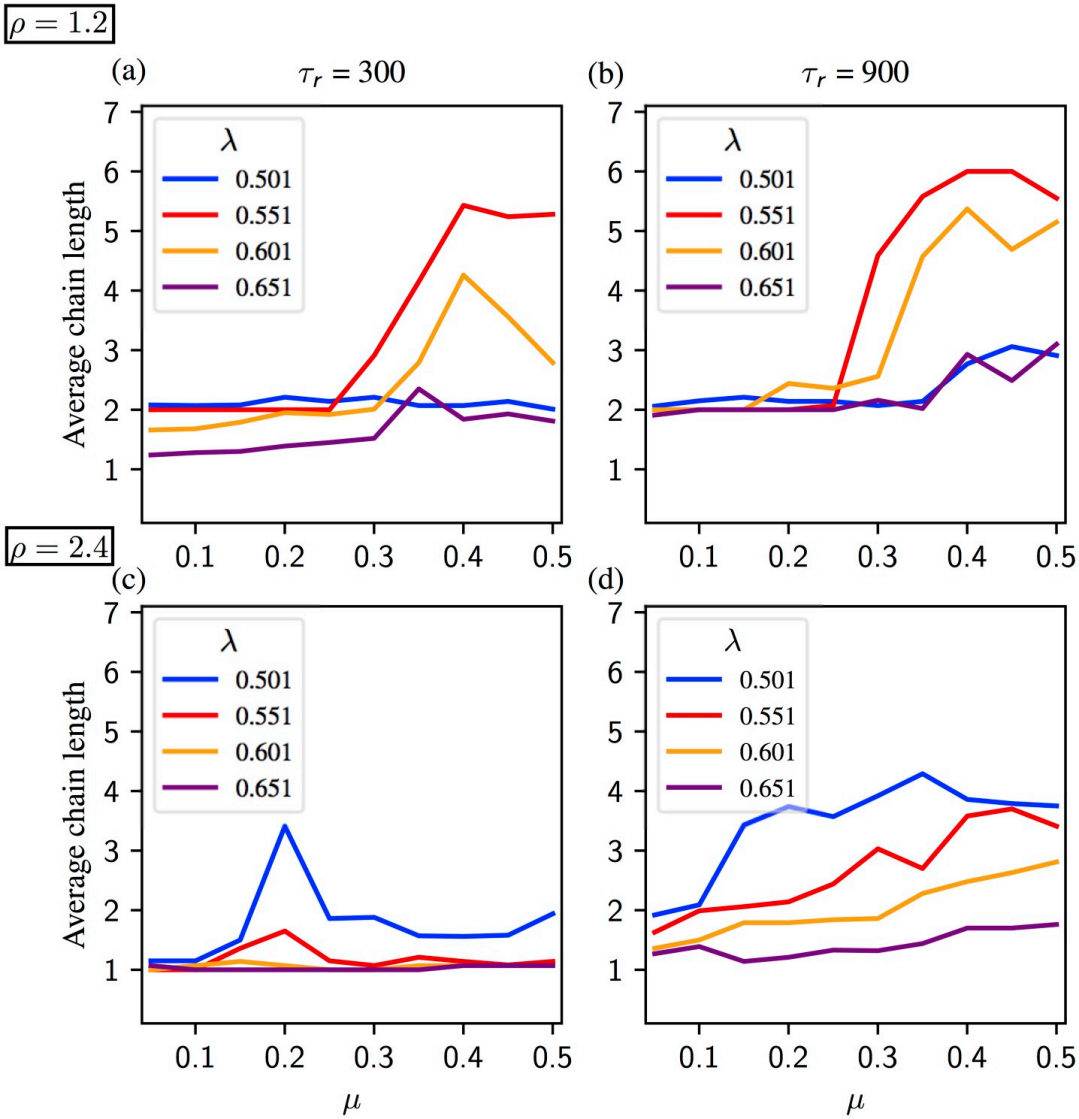
Average chain length in a regular segment for noise *η* = 0.02. Synaptic time constant equals to *τ*_*r*_ = 300 on panels (a) and (c), and *τ*_*r*_ = 900 on panels (b) and (d). (a, b) Activity for *ρ* = 1.2. The chain length increases with *μ* (1/neural gain) and *τ*_*r*_. The global inhibition value, λ, should be high enough for a sequential activation, but the chain length decreases if λ is too high. (c, d) Activity for *ρ* = 2.4. The chain length increases with *μ* and *τ*_*r*_, but decreases with λ. See S1 Fig for the details of the activity.

**Fig 6:**
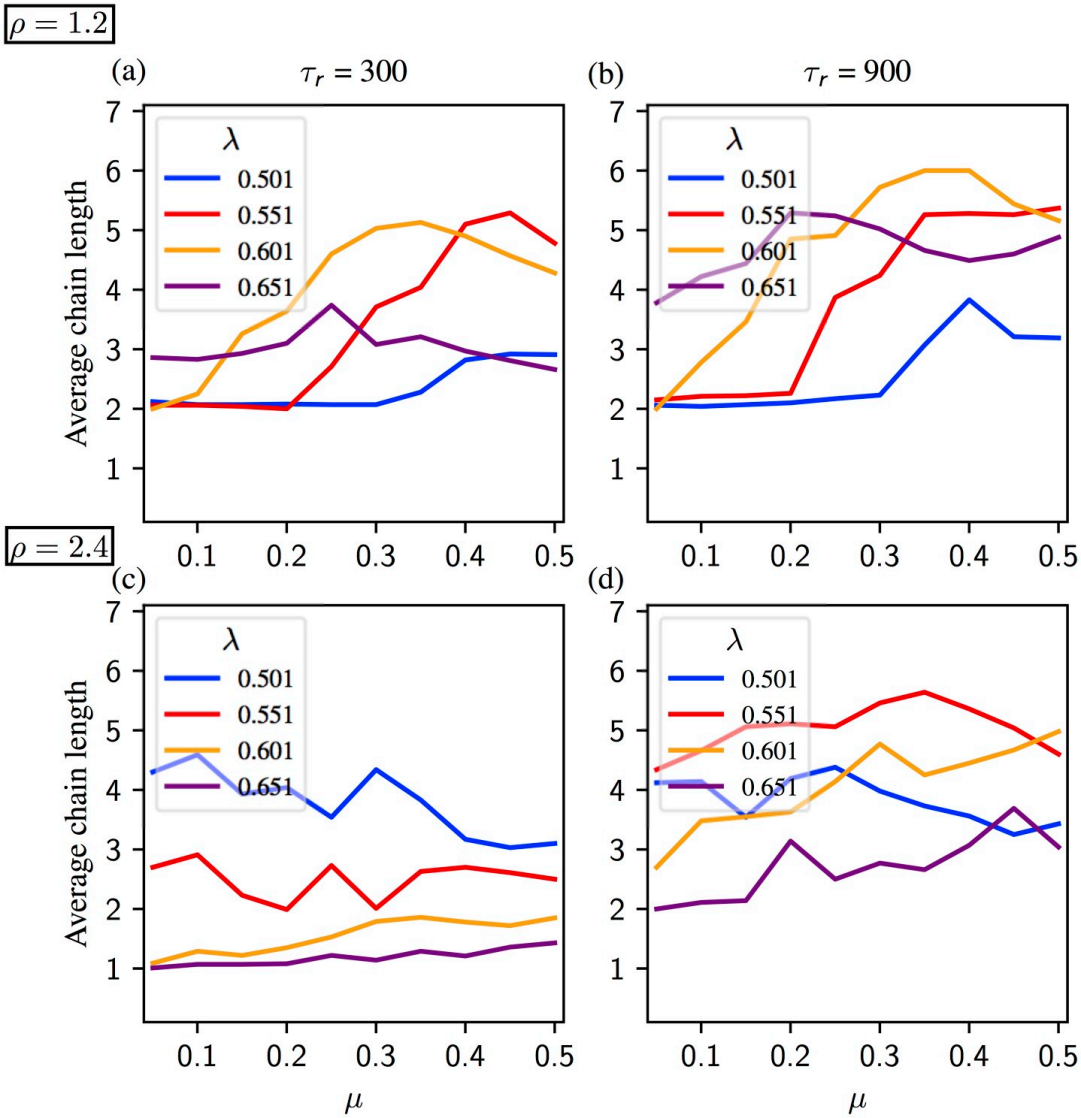
Average chain length in a regular segment for noise *η* = 0.04. Synaptic time constant equals to *τ*_*r*_ = 300 on panels (a) and (c), and *τ*_*r*_ = 900 on panels (b) and (d). (a, b) Activity for *ρ* = 1.2. The chain length increases with *τ*_*r*_. Chains are longer for intermediate values of *μ* (intermediate values of neural gain), but shorter for *μ* too high (low neural gain). Increasing inhibition λ facilitates regular pattern activation for the low values of *μ*, see for instance λ = 0.501 vs λ = 0.601 in (a), but ceases if it is too strong. (c, d) Activity for *ρ* = 2.4. The chain length increases with *τ*_*r*_, but decreases with λ. Increasing *μ* lengthens the chains more with *τ*_*r*_ = 900 than *τ*_*r*_ = 300. See S2 Fig for the details of the activity.

The relation between the model parameters and the sequential activation is more subtle with the learning matrix (4) derived from the most simplified Hebbian learning rule than a structured one as in [40]. In Figs 5 and 6 we present the mean chain length for two different noise intensities; *η* = 0.02 and *η* = 0.04, respectively. In each figure synaptic parameters are *ρ* ∈ {1.2, 2.4},*τ*_*r*_ ∈ {300, 900}. Neural parameters λ and *μ* are varied within a range assuring the existence of chains of at least length 2. Details of the sequences are shown in S1 and S2 Figs. Statistics on chain length show main effects of each parameter (*η* (***), *μ* (**), λ (***), *τ*_*r*_ (***) and *ρ* (***). They also show an interaction between the five parameters altogether (***).

In all panels of Fig 5 where *η* = 0.02, the average chain length increases with *μ*, unless *μ* is too large. In Fig 5a and 5b we observe a sharp increase in the chain length for λ = {0.551, 0.601} (less pronounced for λ = {0.501, 0.651}). The sharp increase in the average chain length occurs when the bifurcation scenario changes around *μ* ≈ μ* (for the definition of μ* see Sec. Analysis of the dynamics and section “Dynamic bifurcation scenarios” in S1 Appendix) While middle range inhibition leads to longer sequences for *ρ* = 1.2, weak inhibition is more suitable for *ρ* = 2.4 (Fig 5c and 5d). Indeed, λ and *ρ* have interacting effects on chain length (***).

In Fig 6, the noise level is increased to *η* = 0.04. Generally speaking, increasing the noise level prolongs the chains by facilitating the activation. Especially for *τ*_*r*_ = 900 and λ = 0.651, the average chain length is considerably higher with *η* = 0.04 (Fig 6) than *η* = 0.02 (Fig 5). On the other hand, the relation between the average chain length and *μ* becomes more delicate. In Fig 6a and 6b for *ρ* = 1.2, the average chain length peaks for the intermediate values of *μ*. Strong inhibition λ = 0.651 prolongs the chains for small values of *μ* and slow synapses, whereas intermediate values of inhibition λ = {0.551, 0.601} favor longer chains as *μ* increases. For *ρ* = 2.4 (Fig 6c and 6d) chains are longer under weak inhibition λ = 0.501. However, increasing *μ* under weak inhibition considerably shortens the chains. The average chain length increases with *μ* under strong inhibition for λ = {0.601.0.651} Parameters λ and *μ* have interacting effects on chain length (***).

Our analysis unveils a nonlinear relation between *μ* and the chain length. When *μ* is small, an increase of *μ* provokes an increase of the length of the chain. However, in most cases we find that the chain lengths are maximal for intermediate values of *μ*. This is clear intuitively: large gain (small *μ*) prevents the units from deactivating, making the transition from one pattern to the next difficult. Small gain, on the other hand, prevents the next unit from activating. Another factor is the occurrence of the transition from scenario 1 to 2 (see Sec. Analysis of the dynamics and section “Dynamic bifurcation scenarios” in S1 Appendix). The synaptic product *ρ* and the global inhibition parameter λ also influence the system’s behaviour. For *ρ* = 1.2, inhibition in the middle range leads to longer sequences, whereas weak inhibition is more suitable for *ρ* = 2.4.

### Analysis of the dynamics

Latching dynamics is defined as a sequence (chain) of activations of learned patterns that de-activate due to a slow process (e.g., adaptation, here synaptic depression), allowing for a transition to the next learned pattern in the sequence [11, 75]. Here we refine this description using the language of dynamics and multiple timescale analysis. The main idea is to treat the synaptic variables *s*_*i*_ as *slowly varying parameters*, so that the evolution of the system becomes a *movie* of the dynamical configurations of the units *x*_*i*_. On the other hand the firing rate Eq (1) is well adapted to analyze latching dynamics. Indeed, from the form of Eq (1) (assuming for the moment that noise is set to 0) one can immediately see that whenever *x*_*i*_ is set to 0 or 1, this variable stays fixed at any time. Therefore considering any face in the hypercube [0, 1]^*N*^ defined by two coordinates (*x*_*i*_, *x*_*j*_), the other coordinates being fixed at 0 or 1, it is invariant under the flow of Eq (1). In other words, any trajectory starting on the face stays entirely on it. This is of course true also for the edges and vertices at the boundary of each face. Each vertex is an equilibrium of Eq (1) and connections between such equilibria can be realised through edges of the hypercube, which greatly simplifies the analysis.

When the couple (*x*_*i*_, *s*_*i*_) of unit *i* is set at (1, 1), *x*_*i*_ is fixed as we have seen but STD Eq (3) induces an asymptotic decrease of the synaptic variable towards the value *S* = (1 + *ρ*)^−1^. This in turn weakens the synaptic weight *J*^*max*^ *s*_*i*_ in Eq (1), which may destabilize *ξ*^*i*^ in the direction of ${\hat{\xi }}^{i}$. Considering *s*_*i*_ as a slowly varying parameter this can be seen as a *dynamic bifurcation* of an equilibrium along the edge from *ξ*^*i*^ to ${\hat{\xi }}^{i}$. The following scenario was described in [40]. For the sake of simplicity we now assume *i* = 1 (the same arguments hold for any *i*). The patterns *ξ*^1^, ${\hat{\xi }}^{1}$ and *ξ*^2^ lie at the vertices of a face, which we call *Φ*, generated by the coordinates *x*_1_ and *x*_3_, with *x*_2_ = 1 and the rest of the coordinates being set to 0.

**Fig 7:**
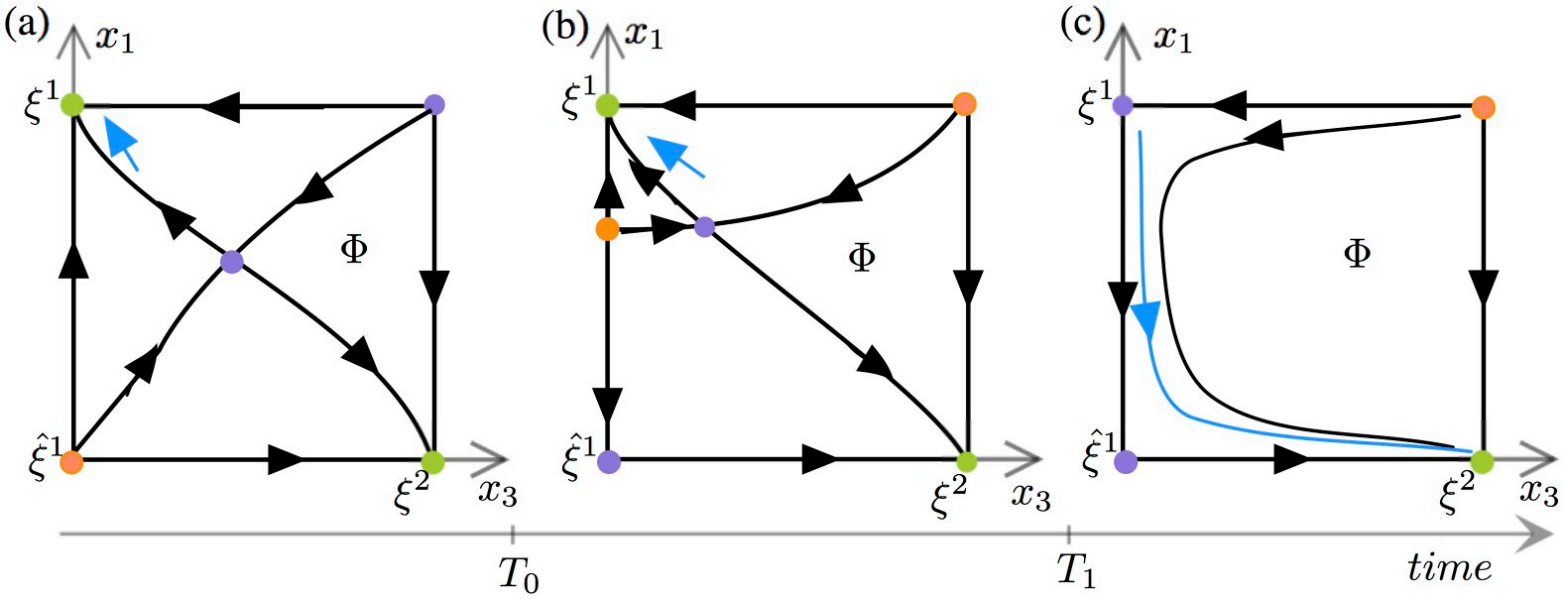
Representative phase portraits of the fast dynamics on the face *Φ* corresponding to the scenario of [40]. The phase portraits are shown at three different ‘slow’ STD times. Green, orange and purple dots represent the stable, completely unstable and saddle equilibria, respectively. The blue lines illustrate segments of trajectory starting near *ξ*^1^. (a) The learned patterns *ξ*^1^ and *ξ*^2^ are stable for *t* < *T*_0_. (b) The bifurcation of a saddle point on the edge between *ξ*^1^ and ${\hat{\xi }}^{1}$ happens for *T*_0_ < *t* < *T*_1_. (c) Pattern *ξ*^1^ has become unstable along the edge $\xi^{1}-{\hat{\xi }}^{1}$ whilst *ξ*^2^ is still stable for *t* > *T*_1_. The saddle point in the interior of *Φ* merges with the bifurcated equilibrium before (c) is realised.

Fig 7 shows three successive snapshots of the movie on *Φ*. The left panel illustrates the initial configuration, with the stable pattern *ξ*^1^ corresponding to the top left vertex. Then at some time *T*_0_ an equilibrium bifurcates out of ${\hat{\xi }}^{1}$ in the direction of *ξ*^1^ (here the ‘slow’ STD time plays the role of bifurcation parameter, see middle panel). After a time *T*_1_ (right panel) this bifurcated equilibrium disappears in *ξ*^1^ which becomes unstable and a connecting trajectory is created along the edge with ${\hat{\xi }}^{1}$. Simultaneously a trajectory connects ${\hat{\xi }}_{1}$ to *ξ*_2_ along the corresponding edge. It results that the following sequence of connecting trajectories is created: $\xi^{1}\to {\hat{\xi }}^{1}\to \xi^{2}$. As a result, any state of the system initially close to *ξ*^1^ will follow the ‘vertical’ edge towards ${\hat{\xi }}^{1}$, then the ‘horizontal’ edge towards *ξ*_2_. The process can repeat itself from *ξ*^2^ to *ξ*^3^ and so on. It was shown in [40] that in order to work, this scenario requires that the coefficients of the matrix *J*^*max*^ satisfy the relation $J_{1,2}^{max}<J_{2,3}^{max}$ (more generally $J_{i,i+1}^{max}<J_{i+1,i+2}^{max}$, *i* = 1, …, *P* − 1, for the existence of a chain of *P* patterns), a condition which does not hold with Eq (4).

The results of this paper rely on the observation that the existence of the connections $\xi^{1}\to {\hat{\xi }}^{1}\to \xi^{2}$ for *t* > *T*_1_ (right panel of Fig 7) is not needed for the occurrence of chains. We will show below that for the connectivity matrix *J* given by Eq (4) the connections exist for at most a unique value of *t* = *T*_1_ and yet regular chains or segments can occur. For *t* > *T*_1_ the connecting trajectory along the edge ${\hat{\xi }}^{1}-\xi^{2}$ is broken by a sink (stable equilibrium) close to ${\hat{\xi }}^{1}$. In such case strong enough noise perturbations could push a trajectory out of the basin of attraction of the sink to the basin of attraction of *ξ*^2^. As a result the trajectory would get past ${\hat{\xi }}^{1}$ and converge towards *ξ*^2^, as expected. When such chains driven by noise exist, we call them *excitable chains* by reference to [57] who introduced the concept. In the case when the connections exist for *t* = *T*_1_ chains occur with noise of arbitrarily small amplitude, because as *t* approaches *T*_1_ from above the amplitude of noise needed to jump over to the basin of attraction of *ξ*^2^ converges to 0. We extend the terminology *excitable chain* to this case also.

**Fig 8:**
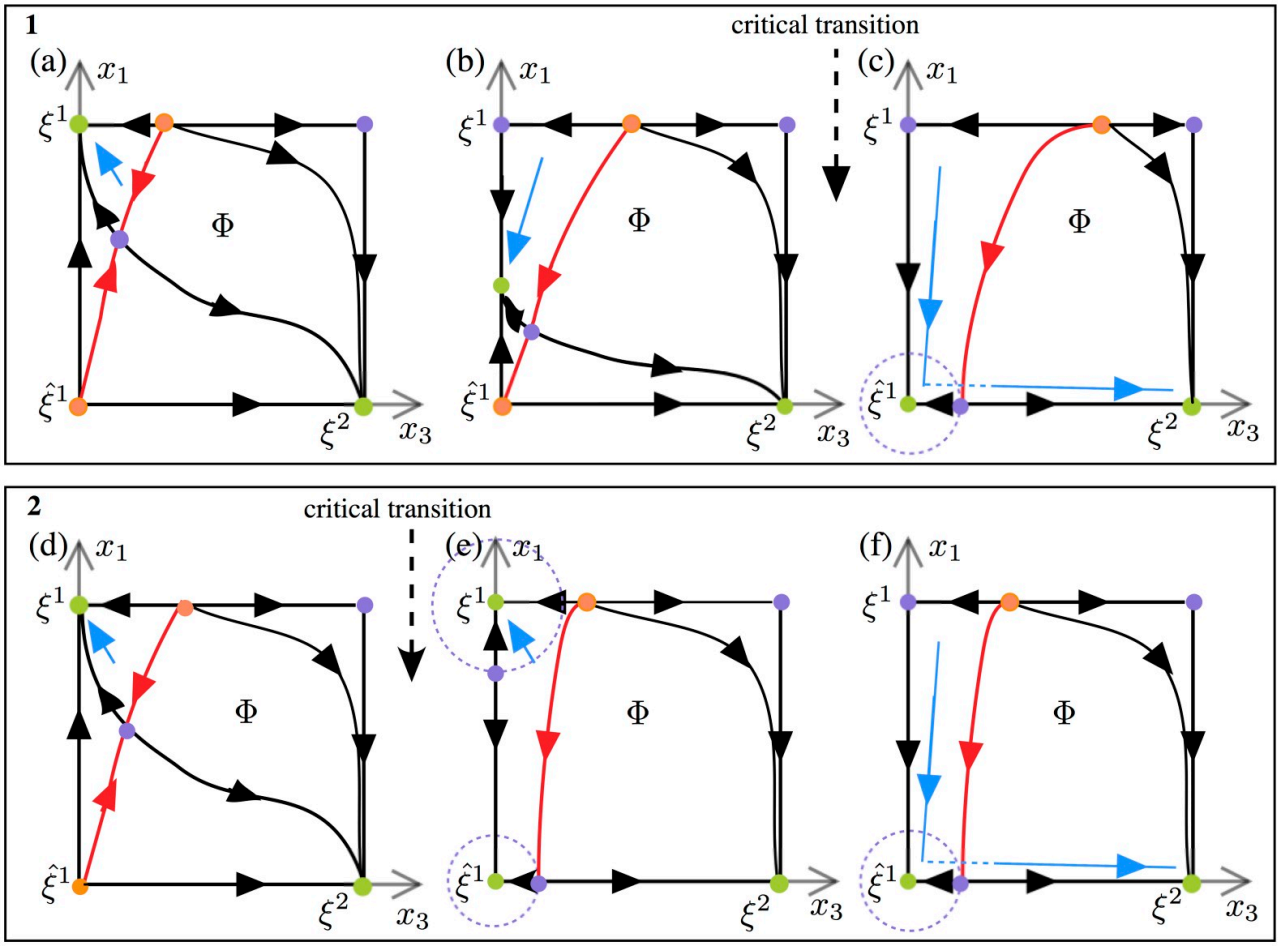
Phase portraits of the fast dynamics on the face Φ at three different ‘slow’ STD times. Stable patterns are coloured in green. The red trajectories are separatrices between the basins of attraction of the stable equilibria. The blue lines illustrate segments of a trajectory starting near *ξ*^1^. Box (1) panels (a,b,c) Phase portraits illustrate the mechanisms that can lead to transition *ξ*^1^ → *ξ*^2^ with excitable connections in Scenarios 1 as time evolves. (a) Trajectory starting near *ξ*^1^ converges to *ξ*^1^. (b) Trajectory follows the saddle between *ξ*^1^ and ${\hat{\xi }}^{1}$ on the *x*_1_-axis. (c) Trajectory “jumps” out of the basin of attraction of ${\hat{\xi }}^{1}$ under the effect of noise and converges towards *ξ*^2^ to *ξ*^1^. Box (2) panels (d,e,f) Phase portraits illustrate the mechanisms that can lead to transition *ξ*^1^ → *ξ*^2^ with excitable connections in Scenarios 2 as time evolves. (d) Trajectory starting near *ξ*^1^ converges to *ξ*^1^. (e) Trajectory remains in the basin of attraction of *x*^1^ defined by the saddle on the *x*_1_-axis. (f) Trajectory “jumps” out of the basin of attraction of ${\hat{\xi }}^{1}$ under the effect of noise and converges towards *ξ*^2^.

Under this new scheme of excitable chains the number of possible transitions is much larger and multiple outcomes are possible. We have identified two scenarios (named 1 and 2) by which these excitable chains can occur in our problem. Typical cases are illustrated on Fig 8. As in Fig 7, snapshots of the dynamics at three different “slow” times are shown. The red line marks the boundary of the basin of attraction of *ξ*^2^ and the dashed circles mark the closest distances for a possible stochastic jump out of it. In both scenarios a completely unstable equilibrium point exists on the edge from *ξ*^1^ to the unnamed vertex on *Φ*, which corresponds to the pattern (1, 1, 1, 0, …, 0) (not a learned pattern).

**Fig 9:**
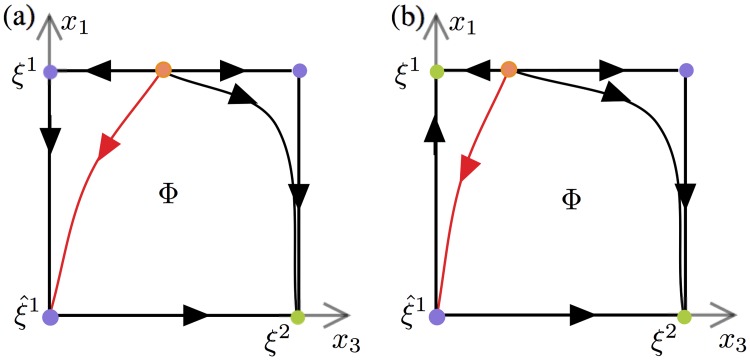
Phase portraits corresponding to critical transitions in Scenario 1 and Scenario 2. These phase portraits occur for a special value of *σ* such that ${\hat{\xi }}^{1}$ changes from a saddle to a sink. (a) The phase portrait of the critical transition corresponding to Scenario 1 contains a sequence of connecting trajectories from *ξ*^1^ to *ξ*^2^, allowing for a transition from *ξ*^1^ to *ξ*^2^ with arbitrarily small noise (e.g. for *ρ* = 2.4, *τ*_*r*_ = 900, λ = 0.55, *μ* = 0.45, the critical transition occurs at *s*_1_ ≈ 0.45, *s*_2_ ≈ 0.56). (b) In the phase portrait of the critical transition corresponding to Scenario 2, a transition from *ξ*^1^ to *ξ*^2^ would fail unless the noise amplitude is sufficiently large. (e.g. for *ρ* = 2.4, *τ*_*r*_ = 900, λ = 0.55, *μ* = 0.15, the critical transition occurs at *s*_1_ ≈ 0.37, *s*_2_ ≈ 0.49).

Under Scenario 1 the pattern *ξ*^1^ first loses stability by a dynamic bifurcation of a sink (stable equilibrium) along the edge $\xi^{1}-{\hat{\xi }}^{1}$. The trajectory, which was initially in the basin of attraction of *ξ*^1^, follows this sink while it is traveling along the edge $\xi^{1}-{\hat{\xi }}^{1}$ towards ${\hat{\xi }}^{1}$ (Fig 8b). In this time interval the distance between the sink and the attraction boundary of *ξ*^2^ (the red line in Fig 8b) is decreasing. Hence it becomes more likely for the noise to carry the trajectory over the *ξ*^2^ stability boundary, activating a transition to *ξ*^2^. The noise level necessary for the jump becomes smaller as the sink approaches ${\hat{\xi }}^{1}$. The critical transition shown in Fig 9a occurs as the sink reaches ${\hat{\xi }}_{1}$. At this moment, noise of arbitrarily small amplitude can cause the transition. Subsequently ${\hat{\xi }}_{1}$ is transiently stable and the distance to the *ξ*^2^ attraction boundary increases. Therefore, it becomes likely that the trajectory remains trapped in the basin of attraction of ${\hat{\xi }}^{1}$, as shown in Fig 8c). A further decrease of *s*_2_ occurring with the passage of time gives a loss of stability of ${\hat{\xi }}^{1}$ and the trajectory leaves Φ to the inactive state. This is the mechanism of the termination of the regular part of the chain (see Fig 2 for an example).

In Scenario 2 the overlap equilibrium point ${\hat{\xi }}^{1}$ becomes a stable before *ξ*^1^ loses stability. At the critical transition (Fig 9b), a sequence of connections from *ξ*^1^ to *ξ*^2^ does not exist, hence the trajectory cannot pass from *ξ*^1^ to *ξ*^2^ unless the noise is sufficiently large. In the context of this scenario regular chains tend to be substantially shorter.

As we have described above, noise is indispensable for crossing the attraction boundary of the next pattern, hence it is crucial for the chains we study. Minimum noise level required for jumps is scenario dependent. In Scenario 1, as *s*_1_ and *s*_2_ decrease along the trajectory, the distance between the sink and the *ξ*^2^ attraction boundary also decreases, becoming arbitrarily small as the critical transition is approached. Thus the noise amplitude required for a jump also decreases to 0. In Scenario 2 noise needs to be stronger to make the trajectory cross over the excitability thresholds of *ξ*^1^ and ${\hat{\xi }}^{1}$. On the other hand, we should also keep in mind that too strong noise can hinder regular chains. Simulations (and analysis, see S1 Appendix) identify *μ* as the main control parameter which determines the choice between these scenarios: the system follows Scenario 1 for higher values of *μ* and Scenario 2 for lower values of *μ*. This explains the difference in behavior seen in Fig 4 at lower and higher values of *μ*. The boundary between the two regions is defined by the value *μ* = μ* for which *ξ*^1^ and ${\hat{\xi }}^{1}$ change stability at the same time. For an analytic definition of μ* and more detailed analysis, see S1 Appendix. In the next section we will see how these scenarios affect irregular activation.

### Irregular chains and additional numerical results

The question we address here is what happens after the last pattern of a regular segment has been reached. We let (*i*, *i* + 1) denote the last pattern of the regular sequence. It follows from previous analysis that the trajectory will remain near ${\hat{\xi }}^{i}$ for a considerable amount of time. Subsequently ${\hat{\xi }}^{i}$ can lose stability and the trajectory passes to the inactive state, or ${\hat{\xi }}^{i}$ remains stable indefinitely. By our choice of parameters (setting *I* = 0, see Sec. Model) the inactive state is marginally stable, which means that an irregular activation will eventually happen due to small noise and a the next pattern will be chosen at random. The mechanism of random activation from ${\hat{\xi }}^{i}$, is similar, with the exception that chain reversal is likely to occur in this case. The transition time to an irregular activation may be significantly longer than in the case of regular transitions, which allows for the recovery of the synaptic variables. Table 1 assembles the features of regular and irregular activations, as predicted by the analysis.

**Table 1:** Features of regular and irregular transitions as predicted by the analysis of Sec. Analysis of the dynamics.

Cognitive function	Regularity	Dynamic mechanism
Predicted sequence of activation of learned patterns	Deterministic behaviour	Dynamic bifurcation, mostly scenario 1, computable transition time by integration
Irregular activation of learned patterns	Non-uniform probability distribution (Fig 12, S3 and S4 Figs)	Transition to a neutrally stable de-activation or overlap state, random transition time

Our numerical results confirm the trends of the analytical predictions, at the same time showing that the dynamics of the model is more complex and other parameters play a very important role. In particular, interestingly, the longest regular segments are observed for *μ* values corresponding to scenario 1 close to *μ* = μ*, which is the boundary value separating scenarios 1 and 2 (see Sec. Analysis of the dynamics). Regular segments typically become shorter if *μ* is increased significantly beyond μ*, see Figs 5 and 6. This means that there exists an optimal *μ* window for the existence of long regular segments, or, in other words, neuronal gain needs to be not too small and not too large.

In this section we will discuss features of irregular activation, based on numerical results. To show statistics of irregular activation we define a measure of ‘distance’ Δ, as follows. Suppose at a time *t*, *x*_*p*_ and *x*_*q*_ are the two most recently activated units, with *x*_*p*_ preceding *x*_*q*_ in its activation. We define

$$\begin{array}{c} \Delta =q-p. \end{array}$$

Note that a regular chain satisfies Δ = 1 for all *t* until the last pattern is reached.

We distinguished two cases of irregular continuation of chains: reversing the chain (Δ = −1) and random reactivation of new chains (Δ ≠ −1). We allow the possibility of Δ = 1 as such chains can occur in an irregular way for the following reason: an irregular activation is typically preceded by a complete deactivation, usually with a prolonged passage time. The new activation is random, so that an activation to the pattern which is next in the overlap sequence can occur, for instance the sequence [1, 2]→[2]→[] → [3]→[2, 3]…. Such reactivations are documented in details in S3 and S4 Figs.

Recall the scenarios 1 and 2 for transitions from one pattern to the next (Fig 8). The former occurs for “large” values of *μ* and the latter for lower values of *μ*. Let ${\hat{\xi }}^{p}$ be the last intermediate state at the end of the regular segment. In Scenario 2 either ${\hat{\xi }}^{p}$ remains stable indefinitely or it destabilizes after some (long) time due to the repotentiation of *s*_*p*_. The latter case corresponds to a dynamic scenario for a chain’s reversal. We refer to the prolonged residence of the system at ${\hat{\xi }}^{p}$ as *pending* and note that it likely leads to a reversal. However, for high values of noise, random activation (Δ ≠ −1) may also occur. The scenario 1 is more likely to yield random re-activation as ${\hat{\xi }}^{p}$ loses stability in the *x*_*p*+1_ direction with the decrease of *s*_*p*+1_, so that a transition to the inactive state is possible. Notice that in this case too other Δ values are possible when the noise is large. Statistics on distance of irregular chains show main effects of parameter *η* (*), *μ* (***), λ (*) and *ρ* (**). The four parameters *η* (*), *μ* (***), λ (*) and *τ* also have effects on activation distance and interact with each other (***).

**Fig 10:**
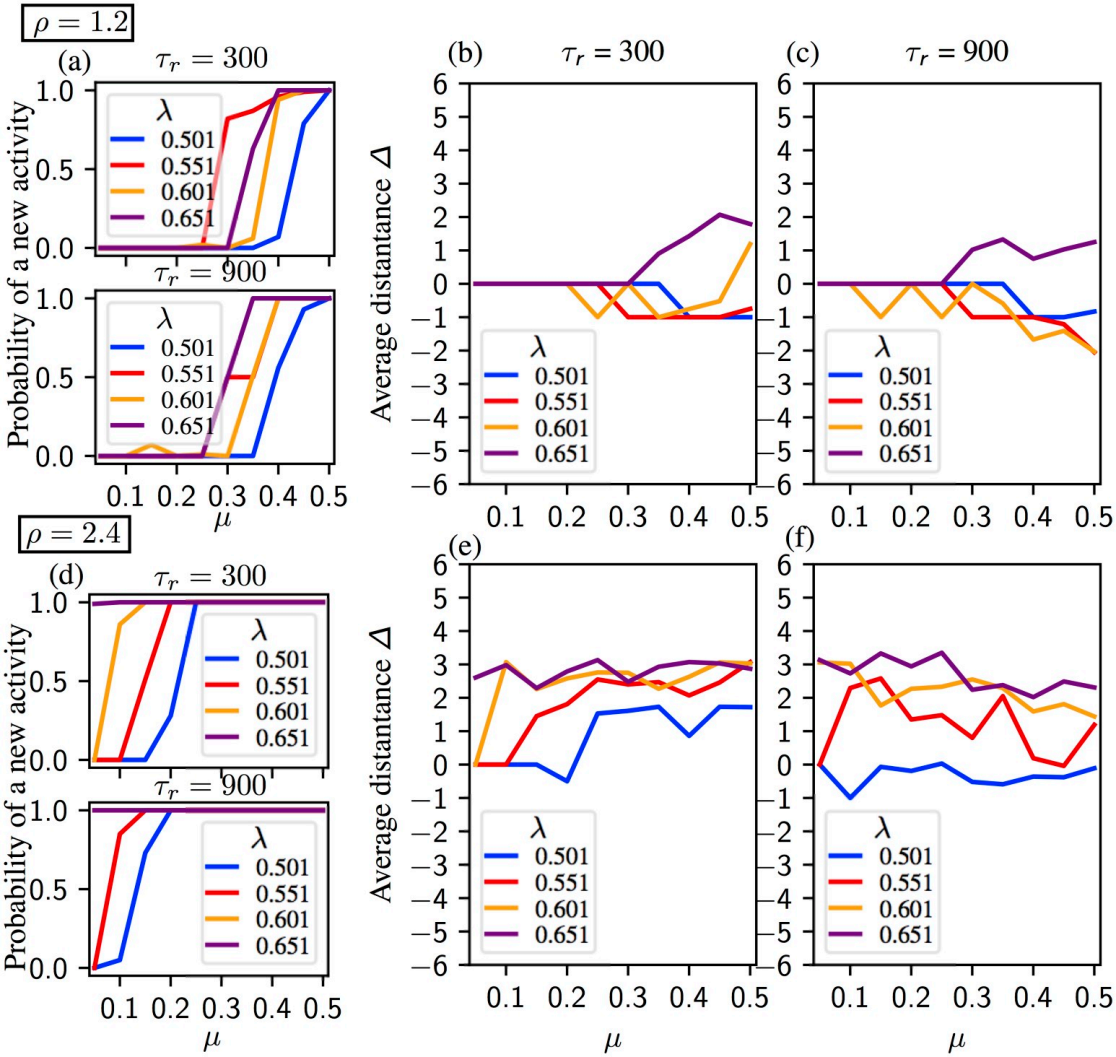
Probability of a new activity and average distance Δ for noise *η* = 0.02. Synaptic time constant equals to *τ*_*r*_ = 300 in panels (b) and (e), and *τ*_*r*_ = 900 in panels (c) and (f). (a, b, c) Activity for *ρ* = 1.2. (a) The probability of a new activity after the initial sequence for *ρ* = 300 (upper panel) and *τ* = 900 (lower panel). Small values of *μ* (1/neural gain) tends to keep the system on the last activated pattern. Minimum *μ* value required for a new sequence decreases with inhibition λ and *τ*_*r*_. (b, c) Activated patterns mostly remain in negative distances except for λ = 0.651 for *τ*_*r*_ = {300, 900}, for high values for *μ* for λ = 0.601 with *τ*_*r*_ = 300. (d, e, f) Activity for *ρ* = 2.4. (d) The probability of a new activity after the initial sequence for *ρ* = 300 (upper panel) and *τ* = 900 (lower panel). Minimum *μ* value required for a new sequence decreases with inhibition λ and time constant *τ*_*r*_. A new activation is always observed for *τ*_*r*_ = 300, λ = 0.651; and for *τ*_*r*_ = 900, λ = {0.601, 0.651}. (e) Average distance Δ is positive and increases with *μ*. (f) Average distance Δ is negative and increases with *mu* for λ = 0.501. Average distance Δ is positive and decreases with *μ* for λ = {0.551, 0.601, 0.651}.

**Fig 11:**
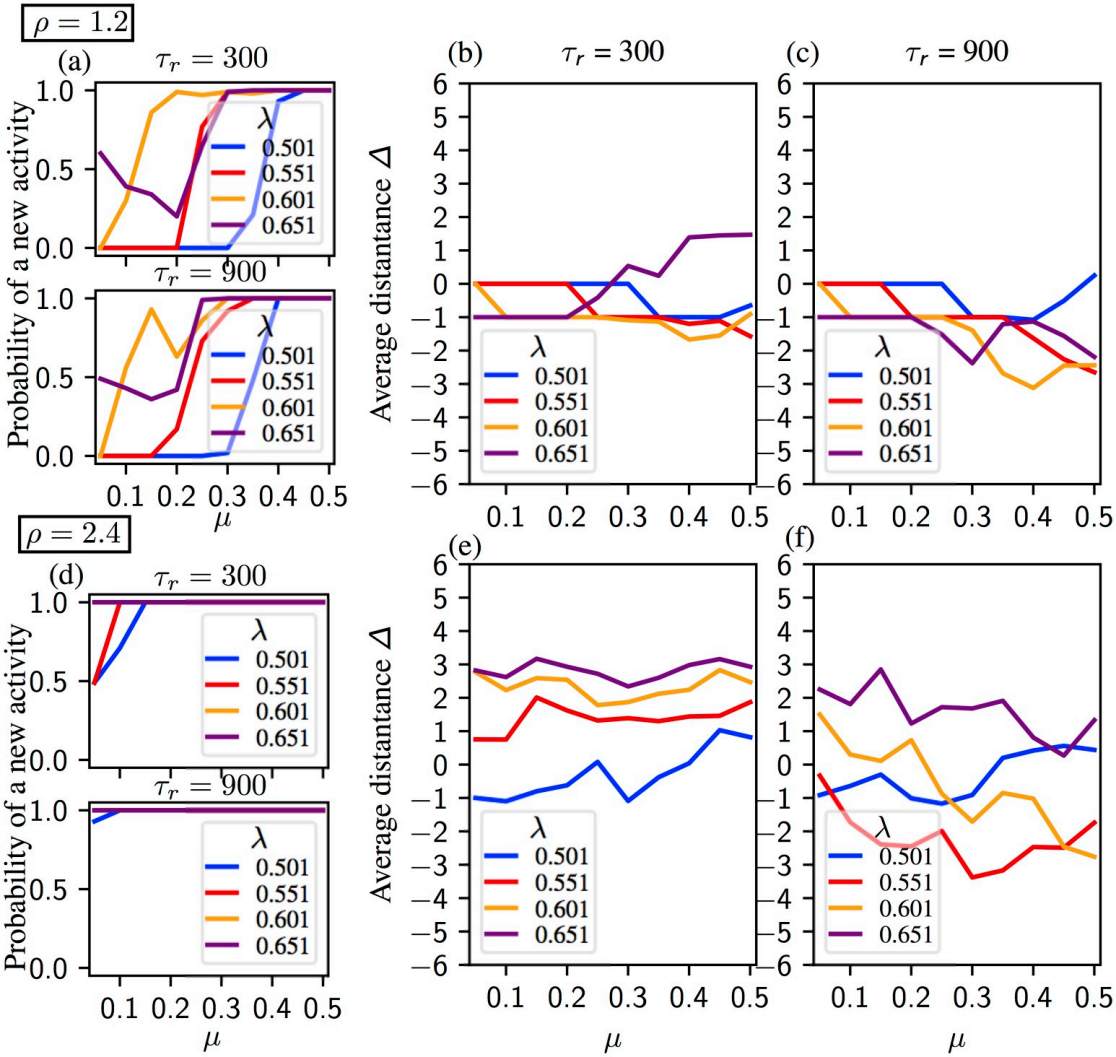
Probability of a new activity and average distance Δ for noise *η* = 0.04. Synaptic time constant equals to *τ*_*r*_ = 300 in panels (b) and (e), and *τ*_*r*_ = 900 in panels (c) and (f). (a, b, c) Activity for *ρ* = 1.2. (a) The probability of a new activity after the initial sequence for *ρ* = 300 (upper panel) and *τ* = 900 (lower panel). Minimum *μ* (1/neural gain) value required for a new sequence decreases with inhibition λ and time constant *τ*_*r*_. (a, b) Average distance is negative except for *μ* > 0.3, λ = 0.651 and *τ*_*r*_ = 300. (d, e, f) Activity for *ρ* = 2.4. (d) The probability of a new activity after the initial sequence for *ρ* = 300 (upper panel) and *τ* = 900 (lower panel). A new activation is always observed for *τ*_*r*_ = 300, λ = {0.601, 0.651}; and in almost all trials for *τ*_*r*_ = 900.(e) Average distance Δ is positive except for λ = 0.501 where it increases from negative to positive with increasing *μ*. (d) Average distance Δ decreases for all cases except for λ = 0.501 where it increases from negative to positive with increasing *μ*.

Figs 10 and 11 show the average activation distance Δ for *η* = 0.02 and *η* = 0.04, respectively. For λ = {0.501, 0.551} and small values of *μ*, the system remains on the last activated pattern. Activity with Δ = −1 is generally supported for *ρ* = 1.2 and for *ρ* = 2.4 if (*μ*, λ) are small. Indeed, λ, *ρ* and *μ* have interacting effects on the activation distance (***). We do not see any activation for small values of *μ* in Fig 10 which indicates that the activity remains either on a pattern *ξ* or on an intermediate state $\hat{\xi }$. Increasing *μ* introduces a backwards activation, except for λ = 0.651 for which the new activity is in the forward direction. For *ρ* = 2.4, we observe that the average distance increases with λ and *μ* if *τ*_*r*_ = 300, but decreases with *μ* if *τ*_*r*_ = 900.

Increasing the noise level *η* (Fig 11) facilitates activation of new patterns. As Fig 11a demonstrates, the system remains on the last activated pattern only for λ = {0.501, 0.551} and small values of *μ*, New activation mostly stays in the negative distance for *ρ* = 1.2 unless for high values λ and *μ* (Fig 11b and 11c). Indeed, there are interactive effects between λ, *η* and *ρ* (**) and between λ, *ρ* and *μ* (**). Taking *ρ* = 2.4 considerable changes the average Δ for both synaptic time constants. Probability of a new activity is above 0.5 for all parameter combinations (Fig 11d). For *τ*_*r*_ = 300, the average stays in the positive region for the whole range of *μ*, unless λ = 0.501 for which average Δ climbs from negative to positive values (Fig 11e). A similar pattern is observed with λ = 0.501 and the synaptic time constant *τ*_*r*_ = 900 (Fig 11f). However, the average Δ decreases with *μ* and for the other values of λ when *τ*_*r*_ = 900.

Supporting materials S3 and S4 Figs show the percentage of Δ values after a new activation for *η* = 0.02 and *η* = 0.04, respectively. Recall that as the chains get longer with increasing *μ*, regular segments get longer, as well, specially when noise is high (*η* = 0.04). Regarding the type of forward and/or backward irregular chains, high values of *ρ* and high values of *μ* (e.g. low gain) for low values of noise *η* (S3 Fig) increase the possibility for irregular chains in the forward direction, while the combination of high values of *μ* (e.g. low gain), *ρ* and noise *η* increase the possibility for irregular chains in both directions (S4 Fig). The difference between the percentages of Δ for *τ*_*r*_ = 300 and *τ*_*r*_ = 900 indicates the capability of slow synapses to yield longer chains.

**Fig 12:**
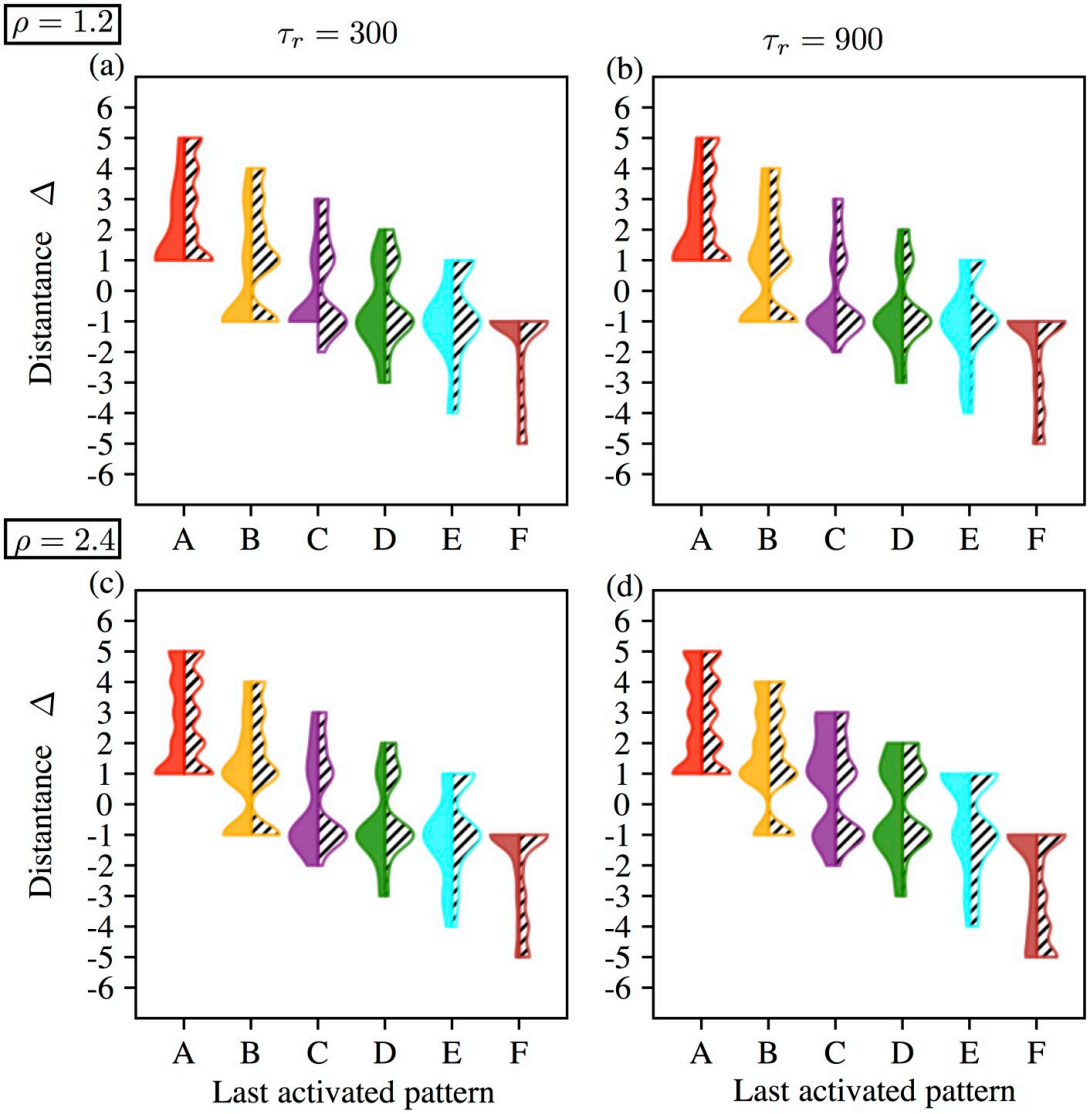
Distribution of activation distance Δ at last activated patterns from A to F over all trials. The distributions are bounded by the minima and maxima of the Δ sets. Each violin is colored with respect to the color code of the last activated units of a pattern in the forward direction as in Figs 1 and 2. Left half of each violin corresponds to activity with noise *η* = 0.02 and the hashed right halves correspond to activity with noise *η* = 0.04. (a) Activity for *ρ* = 1.2, *τ*_*r*_ = 300. (b) Activity for *ρ* = 1.2, *τ*_*r*_ = 900. (a) Activity for *ρ* = 2.4, *τ*_*r*_ = 300. (b) Activity for *ρ* = 2.4, *τ*_*r*_ = 900.

In order to see the relation between the chain length and distance Δ for each combination of (*η*, *ρ*, *τ*_*r*_), we categorized the regular sequences with respect to the last activated pattern. Then, we extracted the Δ values among the trials with activation and obtain a Δ set for a each possible last activated pattern of the sequence ABCDEF. Fig 12 shows the distribution of Δ at patterns from A to F in a violin plot. Left side of each violin shows the results with *η* = 0.02, and right side with *η* = 0.04. We remark at first glance that Δ depends on the chain length. Activation is in the forward direction in short chains whereas it is in the backward direction for long chains (still preserving a preference for Δ = −1). While this is related to being a bounded system (in terms of the system size *N* = 8), for the intermediate patterns, like D, negative and positive values of Δ are almost equally distributed (especially for *ρ* = 2.4, *τ*_*r*_ = 900). We also observe that after passing C, the system activates more and more the units in negative distance Δ ≠ −1. Increasing noise spreads the distribution of Δ, very visibly in *ρ* = 1.2 for patterns A, B; also for pattern F in *ρ* = 2.4, *τ*_*r*_ = 900. Finally, distribution of Δ approximates to a normal distribution for shorter chains.

### Perturbed connectivity matrix

In order to test the robustness of our model, we randomly perturbed the off-diagonal elements of the connectivity matrix (4) while ensuring its diagonally symmetric structure. We consider two levels of perturbations (5% and 10%) and a parameter set for which we obtain a wide range of behaviour with matrix (4) (the parameter set: *η* = 0.04, *ρ* = 1.2, *τ*_*r*_ = {300, 900}). The simulation results are presented in Table 2 and in S5 Fig. Looking at Table 2, we see that the difference in the chain lengths are less than 10%, and in the probability of a new activity is around 15%. Furthermore, these two features follow similar patters to the ones of the regular matrix as S5(a)–S5(d) Fig show. The differences between the average distance Δ values are around 18% for *τ*_*r*_ = 300 and 32% for *τ*_*r*_ = 900. In particular, the difference in Δ increases with *μ* and λ for *τ*_*r*_ = 900 S5(e) and S5(f).

**Table 2:** Relative difference between the features of the unperturbed and irregularly perturbed synaptic matrices.

Feature	τ_r_ = 300		τ_r_ = 900	
	5 % perturbation	10 % perturbation	5 % perturbation	10 % perturbation
Average chain length	4.5%	6.8%	6.8%	8.6%
Probability of a new activity	12%	16%	12%	14%
Average distance Δ	18.5%	18%	34%	30%

## Discussion

Experimental evidence indicates that the brain can either replay the same learned sequence to repeat reliable behaviors [12–16] or generate new sequences to create new behaviors [17–21, 76]. The present research identifies biologically plausible mechanisms that explain how a neural network can switch from repeating learned regular sequences to activating new irregular sequences. To make the problem analytically tractable, the combined effects of the parameters were analyzed on neuronal population firing rates in a simplified balanced network model by use of slow-fast dynamics and dynamic bifurcations. We demonstrated how variations in neuronal gain, short-term synaptic depression and noise can switch the network behavior between regular or irregular sequences for a fixed learned synaptic matrix.

Let us point out that the model we have considered represents a general framework of networks with adaptation, thus is likely to have applications in other fields, such as population dynamics, genetics, game theory, sociology or economics.

### Synaptic matrix

In the present model the overlap had the same number of shared units for all the overlapping populations. This allowed us to show that variable overlap is not a necessary condition for the activation of sequences of populations. A consequence of the constant overlap is that sequences from a stimulus-driven end-point pattern in the sequence (e. g. first pattern A of the sequence) are directional but sequences from a mid-point pattern can go in any of the two possible directions. The model can then generate bi-directional sequences interesting in free recall. Starting from the first pattern A (or G), the sequence ABCDEFG is oriented in one direction (or in the other direction), and starting from a middle pattern e.g. D, the sequence can be oriented in any of the two possible directions. The present model allows for bi-directionnal sequences as well as for new sequences depending on the value of neuronal gain *γ* = *μ*^−1^.

Our model is robust against small perturbations of the connectivity matrix. In other words our results would hold, with small modifications, in a slightly heterogeneous network.

### Regular vs. irregular sequences

Regarding regular sequences, the chain length increases with noise and for combinations of strong STD (high values of *ρ*) and low inhibition, or weak STD (low values of *ρ*) and strong inhibition. Further, for most combinations of noise, STD and inhibition, there is an optimal value of gain that generates the longest chains. The sensitivity of a neuron to its incoming activation varies with changes in its gain [65]. Simulations and analysis show that the neuronal gain (1/*μ*) is a key control parameter that selects the length and type of sequence activated: regular or irregular. Large neuronal gain impairs the deactivation of the units in a pattern and hence makes the transition to the next pattern difficult, and small gain impairs the activation of the next unit and again makes the transition difficult. Consequently there is an optimal window for the gain corresponding to long sequences. Experimental evidence shows that presentations of a given stimulus reproduces the same sequence reliably [14, 16, 22, 77]. The present model can repeat systematic full sequences of activation for some values of the parameters that make the network change patterns in a given order. This ‘reliable’ mode could be well adapted to the reliable reproduction of learned sequences of behaviors.

Regarding irregular sequences, a large neuronal gain leads to the second scenario describing transitions from one pattern to the next (as in Fig 8). According to this second scenario, the last intermediate state of the network at the end of a regular segment can destabilize and leads to a reversal of the sequence. In that case the network activates patterns backward in the reverse order. Further, for high values of noise, Scenario 2 can lead to random activation of patterns in either the forward or backward direction. Such variable sequences are more likely to be generated according to the first scenario that makes possible a transition to another state in the forward or backward direction and that does not necessarily overlap with the current state (forward or backward leaps). Direction of recall has been linked to the stimulation amplitude presence of non-context units in [78]. Our model can generate variable sequences over repetitions of the same triggering stimulus for high values of gain, in line with a *memoryless* system [56] that activates a new pattern in an unpredictable fashion. Behavioral studies indicate that presentation of a triggering stimulus can activate distant items that are not directly associated to it [79]. The generation of new sequences corresponding to the activation of new possibilities [80] and the execution of new information-seeking behaviors such as saccades or locomotor explorations of unknown locations [1] rely on variable internal neural dynamics such as in the medial frontal cortex [81]. This ‘creative’ mode of variable activation not following a given sequence could correspond to a mind wandering mode [19, 82] or divergent thinking involved in creativity [83–86].

### Neuromodulation of the switch between regular and irregular sequences

Neuronal gain is reported to depend on neuromodulatory factors such as dopamine [51, 87–89] involved in reward-seeking behaviors and punishment [90–92]. Dopamine is reported to modulate the magnitude of the activation between associates in memory (priming; [93, 94]) and dopamine induced changes in neuronal gain have been reported to account for changes in activation in memory [52] and for changes in neuronal activity that controls muscle outputs [95]. A novel feature of our network model is that neuronal gain influences the type of sequences that are generated: regular or irregular. Typical computational models of sequence generation reproduce learned sequences [15]. However, if the brain must in some case reproduce systematic behaviors, it must also have the capacity to liberate itself from repetition in order to create new behaviors. The present research shows that the network can exhibit the dual behavior of activating regular or irregular sequences for a given synaptic matrix. The transition depends on biological parameters, in particular on gain modulation. Given that changes in gain change the length of the regular sequence, and that when the regular sequence stops it becomes irregular, the gain controls the regularity of the sequences. The present research sheds light on how the brain can switch between a ‘reliable’ mode and a ‘creative’ mode of sequential behavior depending on external factors such as reward that neuromodulate neuronal gain.

### Fixed versus increasing overlap size

Earlier works [40–42] proved the existence of regular sequences under the assumptions of increasing overlap and synaptic efficacy. In this work we showed that neither of these conditions is needed: regular sequences can exist in the context of equal overlap. It is know that populations of neurons coding for memories, and their overlaps, can vary (due to learning) on time scales that are long in the context of this paper, but still relatively short [96]. Sequences arising through increasing overlap can be understood as learned as opposed to the regular sequences in this paper, that occur merely due to the semantic relation between concepts. Consequently, it is interesting to extend our modelling framework to a setting where overlap could vary on a super-slow timescale.

## Supporting information

S1 Appendix (ZIP)

S1 Fig Percentage of last activated patterns in a regular segment for noise *η* = 0.02. Pattern colours follow to the colour codes of the last activated units in Figs 1–4 (see the legend on the right). The height of each colour on a bar indicates the percentage of the corresponding pattern for a given parameter combination over 100 trials. Synaptic time constant equals to *τ*_*r*_ = 300 on panels (a) and (c), and *τ*_*r*_ = 900 on panels (b) and (d). (a, b) *ρ* = 1.2. The chain length increases with *μ* (decreases with neural gain) and *τ*_*r*_. The global inhibition value, λ, should be high enough for a sequential activation, but the chain length decreases if λ is too high. (c, d) *ρ* = 2.4. The chain length increases with *μ* and *τ*_*r*_, but decreases with λ.
(EPS)

S2 Fig Percentage of last activated patterns in a regular segment for noise *η* = 0.04. Pattern colours follow to the colour codes of the last activated units in Figs 1 and 2 (see the legend on the right). The height of each colour on a bar indicates the percentage of corresponding last activated pattern for a given parameter combination over 100 trials. Synaptic time constant equals to *τ*_*r*_ = 300 on panels (a) and (c), and *τ*_*r*_ = 900 on panels (b) and (d). (a, b) *ρ* = 1.2. The chain length increases with *τ*_*r*_. Chains are longer for intermediate values of *μ* (intermediate values of neural gain), but shorter for *μ* too high (low neural gain). Increasing inhibition λ facilitates regular pattern activation for the low values of *μ*, see for instance λ = 0.501 vs λ = 0.601 in (a), but ceases if it is too strong. (c,d) *ρ* = 2.4. The chain length increases with *τ*_*r*_, but decreases with λ. Increasing *μ* lengthens the chains more with *τ*_*r*_ = 900 than *τ*_*r*_ = 300.
(EPS)

S3 Fig Activity after the initial sequence for noise *η* = 0.02 of the simulations given in S1 Fig. Bars are coloured according to the activation distance Δ (see the legend on the right) and the height of each colour indicates the percentage of the corresponding distance Δ. **(a, b)** Activity for *ρ* = 1.2. (a) Percentage of Δ for *ρ* = 300. (b) Percentage of Δ for *ρ* = 900. New sequences are generated as *μ* (1/neural gain) and inhibition λ increase, with a preference in the backward activity with Δ = −1. Forward activity is possible if both λ and *μ* are high. (c, d) Activity for *ρ* = 2.4. (c) Percentage of Δ for *ρ* = 300. Increasing (*μ*, λ) ensures activation in the forward direction. (d) Percentage of Δ for *ρ* = 900. Backward activation is observed for small values of λ. Increasing (*μ*, λ) ensures activation in the forward direction. Overall, the probability of new (forward) activity is higher in *ρ* = 2.4 than *ρ* = 1.2.
(EPS)

S4 Fig Activity after the initial sequence for noise *η* = 0.04 of the simulations given in S2 Fig. Bars are coloured according to the activation distance Δ (see the legend on the right) and the height of each colour indicates the percentage of the corresponding distance Δ. (a, b) Activity for *ρ* = 1.2. (a) Percentage of Δ for *ρ* = 300. (b) Percentage of Δ for *ρ* = 900. New sequences are generated as *μ* (1/neural gain) and inhibition λ increase, with a preference for backward activity for small values of λ. Activation in distance Δ = −1 has the largest probability. Fast synapses activate in distance Δ > 0 for λ = 0.651 and high values of *μ* while slow synapses activate in distance Δ < 0. (c, d) Activity for *ρ* = 2.4. (c) Percentage of Δ for *ρ* = 300. Increasing (*μ*, λ) ensures activation in the forward direction. (d) Percentage of Δ for *ρ* = 900. Probability of distance being Δ < 1 is considerably high for λ = 0.551 but very small for λ. Overall, the probability of generating new activation is higher for *ρ* = 2.4 than *ρ* = 1.2 and activation in distance Δ < 0 is much higher with *τ*_*r*_ = 900 than *τ*_*r*_ = 300.
(EPS)

S5 Fig Average chain length, probability of a new activity and average distance Δ with irregularly perturbed connectivity matrices. Off-diagonal elements of the connectivity matrix are perturbed by 5% and 10% while keeping the resulting synaptic matrix diagonally symmetric, and for each 10 synaptic matrices are generated. System parameters: Noise *η* = 0.04, synaptic constants *ρ* = 1.2 with *τ*_*r*_ = {300, 900}, global inhibition λ = {0.501, 0.551, 0.601, 0.651}, and *μ* = [0.05, 0.50] (1/neural gain) (100 simulations for each combination). Left row shows the results for *τ*_*r*_ = 300 and right row for *τ*_*r*_ = 900. In each panel bold traces show the results obtained with unperturbed connectivity matrix (4), dotted traces (…) with 5% perturbed connectivity matrices, and dash-dotted traces (−.) with 10% perturbed matrices connectivity matrices (mean over 10 different symmetric matrices for each). (a,b) Average chain length with (4) and perturbed connectivity matrices. (c,d) Probability of new activation with (4) and perturbed connectivity matrices. (e,f) Average distance Δ with (4) and perturbed connectivity matrices.
(EPS)
