# Supplementary material for: Neuronal mechanisms for sequential activation of memory items: Dynamics and reliability
Source: PLoS One. 2020 Apr 16;15(4):e0231165. doi: 10.1371/journal.pone.0231165 (PMC7161983; doi:10.1371/journal.pone.0231165)
Supplement: S1 Appendix — (ZIP) [file pone.0231165.s001.zip › S1_Appendix.pdf]

Neuronal mechanisms for sequential activation of memory items: dynamics and reliability

Elif Köksal Ersöz [1,¤], Carlos Aguilar [2], Pascal Chossat [1,3], Martin Krupa [1,3], Frédéric Lavigne [4]

1 Project Team MathNeuro, INRIA-CNRS-UNS, Sophia Antipolis, France
2 Lab by MANTU, Amaris Research Unit , 950 Route des Colles, Biot, France
3 Université Côte d'Azur, Laboratoire Jean-Alexandre Dieudonné, Nice, France
4 Université Côte d'Azur, CNRS-BCL, Nice, France

¤Current Address: LTSI, INSERM U1099, University of Rennes 1, Rennes, France
*elif.koksal@inria.fr

Appendix

Latching dynamics from the slow-fast view point

Throughout this work we have assumed that the firing rates x_i evolve on a faster time scale than the synaptic variables s_i. This can be formalized by redefining Eq. (1) as a slow-fast system:

$$\dot{x_i} = x_i(1-x_i)\left(-\mu x_i - I - \lambda \sum_{j=1}^{N} x_j + \sum_{j=1}^{N} J_{i,j}^{max} s_j x_j\right) + \eta \quad \text{(S1)}$$

$$\dot{s_i} = \varepsilon((1-s_i) - \rho s_i x_i) \quad \text{(S2)}$$

where $\varepsilon = 1/\tau_r$ and $\rho = \tau_r U$ is the parameter introduced in Sec. Model. To keep our presentation consistent with [1] we use a slightly more general version of the model, adding the parameter I that can be understood as feedforward inhibition or as modulation of the excitability of the ith unit. Using the formulation Eq. (S1) we can apply the tools of the slow-fast systems' theory to gain insight into the transitions between learned patterns. Here we will make some basic observations, leaving rigorous slow-fast analysis for a later publication.

Fast subsystem dynamics in the 2D transition plane

By setting $\varepsilon = 0$ in Eq. (S1), we obtain the fast subsystem of Eq. (S1) where s_i's are considered as parameters. This underlies the idea of dynamic bifurcation: as the s_i's change, the features of the dynamics of the fast system, in particular the stability properties of the patterns ξ_i, evolve. We keep in mind, however, that the s_i's must follow the slow flow, so their values are not arbitrary and are related to each other. We are particularly interested in transitions from ξ_i to ξ_{i+1}, with the dynamics passing near $\hat{\xi}_i$. During this transition x_i moves from near 1 to near 0, x_{i+1} stays close to 1, and x_{i+2} moves from near 0 to near 1. The remaining units stay near 0. Hence the relevant dynamics is approximated by the restriction of the fast system to the plane :

$$F^{i+1} = \{(x_1, \ldots, x_N) \ : \ x_{i+1} = 1, \ x_j = 0 \text{ if } j \neq \{i, i+2\}\},$$

given by:

$$\begin{aligned}\dot{x}_i &= x_i(1-x_i)\left(-\mu x_i - I - \lambda(1+x_i+x_{i+2}) + 2s_i x_i + s_{i+1}\right)\\ \dot{x}_{i+2} &= x_{i+2}(1-x_{i+2})\left(-\mu x_{i+2} - I - \lambda(1+x_i+x_{i+2}) + 2s_{i+2}x_{i+2} + s_{i+1}\right).\end{aligned} \tag{S3}$$

Since $0 \le x_i \le 1$ we are interested in the dynamics of Eq. (S3) restricted to the square $[0,1]^2$, whose edges are invariant for the dynamics. The equilibrium points $(x_{i+2}, x_i) = (0,1)$ and $(x_{i+2}, x_i) = (1,0)$ represent ξ^i and ξ^{i+1}, respectively, while $(x_{i+2}, x_i) = (0,0)$ represents $\hat{\xi}^i$ (the relevant picture is given in Fig. (8) in the main part of the manuscript, with the subscripts 1 and 2 replaced by i and $i+1$).

Dynamic bifurcation scenarios

As s_i and s_{i+1} vary in $[S,1]$, where $S = (1+\rho)^{-1}$, the equilibria of Eq. (S3) undergo several bifurcations that are responsible for the transition $\xi^i \to \hat{\xi}^i \to \xi^{i+1}$, which, in the particular context of Eq. (S3), occurs when the eigenvalues of ξ_i or $\hat{\xi}_i$ corresponding to the edge $x_{i+2} = 0$ pass through 0. These eigenvalues can be easily read off from Eq. (S3):

$$\begin{aligned}\sigma_i &= -(\mu + 2\lambda + I) + 2s_i + s_{i+1}\\ \hat{\sigma}_i &= -(\lambda + I) - s_{i+1}.\end{aligned} \tag{S4}$$

Note that both σ_i and $\hat{\sigma}_i$ are time dependent and vary on the same time scale as the s's. According to the above we are interested in the following bifurcations:

- The initially stable equilibrium point $(x_{i+2}, x_i) = (0,1)$ becomes unstable at

  $$2s_i(t_{(0,1)}) + s_{i+1}(t_{(0,1)}) = \mu + 2\lambda + I,$$

  where $t_{(0,1)}$ is the corresponding bifurcation moment.

- Initially unstable equilibrium point $(x_{i+2}, x_i) = (0,0)$ becomes stable at

  $$s_{i+1}(t_{(0,0)}) = \lambda + I,$$

  where $t_{(0,0)}$ is the corresponding bifurcation moment.

The order of these bifurcations depends on the values of μ, λ and I and we identified two different dynamic bifurcation scenarios: Scenario 1 if $t_{(0,1)} < t_{(0,0)}$ and Scenario 2 if $t_{(0,0)} < t_{(0,1)}$.

The phase portraits defining Scenario 1 are sketched in box 1 of Fig 8 and can be characterized as follows:

- Phase portrait (a) in box 1 corresponds to the situation where $\sigma < 0$ and $(0,1)$ is stable. At the same time $\hat{\sigma} > 0$ and $(0,0)$ is unstable.
- Phase portrait (b) in box 1 corresponds to the case $\sigma_i > 0$ and $\hat{\sigma}_i > 0$. In this case $(0,1)$ has become unstable and there exists a stable equilibrium on the x_i-axis, $(x_{i+2}, x_i) = (0, x_i^*)$. The point $(0,0)$ is still unstable.
- $\sigma_i > 0$ and $\hat{\sigma}_i < 0$. In this case $(0,0)$ becomes stable and there exists a saddle at $(x_{i+2}^*, 0)$. There is a possibility of an *excitable* connection, when trajectories travel down to $(0,0)$, jump over to $(x_{i+2}^*, 0)$ by the action of noise, and continue on to $(1,0)$.

The phase portraits defining Scenario 2 are sketched in box 2 of Fig 8 and can be characterized as follows:

- Phase portrait (d) in box 2 corresponds to the situation where $\sigma_i < 0$ and $(0,1)$ is stable. At the same time $\hat{\sigma} > 0$ and $(0,0)$ is unstable.
- Phase portrait (e) in box 2 corresponds to the case when both σ_i and $\hat{\sigma}_i$ are negative. In this case $(0,1)$ is still stable, while $(0,0)$ has become also stable and there exists a saddle type equilibrium on the x_i-axis, $(x_{i+2}, x_i) = (0, x_i^*)$.
- Phase portrait (e) in box 2 corresponds to the case $\sigma_i > 0$ and $\hat{\sigma}_i < 0$. In this case $(0,1)$ has become unstable and trajectories can pass from $(1,0)$ to $(0,0)$ and jump over a saddle point on the line $x_i = 0$, making an excitable connection to $(0,1)$.

The feature that distinguishes Scenarios 1 and 2 is that the saddle $(0, x_{i+2}^*)$ present in Scenario 1 bifurcates dynamically from $(0,0)$. Hence the jump required is arbitrarily small.

Near the bifurcation of $(0,0)$ at $\hat{\sigma}_i = 0$, $s_{i+1} = I + \sigma$ and $s_{i+2} \approx 1$, whereas the value of s_i is harder to compute, but we know that

$$S < s_i < s_{i+1} = I + \lambda.$$

Note that when $\hat{\sigma} = 0$ (equivalently $s_{i+1} = \lambda + I$), the equilibrium at $(0,0)$, corresponding to $\hat{\xi}^i$ for the original system, has two 0 eigenvalues.

We now determine $\mu = \mu^*$ which separates Scenario 1 from Scenario 2. Note that μ^* is defined by the requirement $t_{(0,0)} = t_{(1,0)}$. We will count the time t starting with the previous transition $\hat{\xi}_{i-1} \to \xi_i$ and we will assume that this transition is instantaneous. Note that at that time $s_{i+1} \approx 1$ (this holds for any μ). Given that $s_{i+1}(t_{(0,0)}) = \lambda + I$ we obtain, using the slow equation:

$$t_{(0,0)} = \int_1^{\lambda+I} \frac{ds}{\varepsilon(1 - s(1+\rho))}.$$

Using the assumptions $t_{(0,0)} = t_{(1,0)}$ and $\hat{\sigma}_i(t_{(1,0)}) = 0$ we obtain $2s_i(t_{(0,0)}) = \lambda + \mu$. Given that the computation of s_i is independent of i we now that $s_i = \lambda + I$ at the time of the transition $\hat{\xi}_{i-1} \to \xi_i$. Hence

$$t_{(0,0)} = t_{(1,0)} = \int_{\lambda+I}^{\frac{\mu+\lambda}{2}} \frac{ds}{\varepsilon(1 - s(1+\rho))}$$

This implies that μ^* is given by the following equation:

$$\int_1^{\lambda+I} \frac{ds}{\varepsilon(1 - s(1+\rho))} = \int_{\lambda+I}^{\frac{\mu^*+\lambda}{2}} \frac{ds}{\varepsilon(1 - s(1+\rho))} \tag{S5}$$

Decreasing μ from μ^* gives $t_{(1,0)} > t_{(0,0)}$, i.e. Scenario 2 and increasing μ from μ^* gives $t_{(0,0)} > t_{(1,0)}$, i.e. Scenario 1.

There are some additional constraints that are shared between the two scenarios. First, we require that $\hat{\xi}^i$ should be stable in the transverse directions, in particular in the direction of x_{i+1}. The relevant eigenvalue is $\hat{\sigma}_{i+1} = \mu + I + \lambda - 2s_{i+1}$, which gives, upon substitution of $s_{i+1} = I + \lambda$, $\hat{\sigma}_{i+1} = \mu - I - \lambda$. This introduces another condition:

$$\mu < \lambda + I. \tag{S6}$$

Second, as in [1], we require the stability of ξ^i in the absence of synaptic depression and the stability of $\hat{\xi}^i$ in transverse directions. This implies:

$$I + 2\lambda + \mu < 2, \quad I + \lambda < 1 < I + 2\lambda. \tag{S7}$$

Fig S1. Variation of μ^* versus λ for $\rho = \{1.2, 1.8, 2.4\}$. The function $\mu^*(\lambda)$ changes non-monotonically for $\rho = \{1.2, 1.8\}$ with minima at $(\lambda, \mu) = (0.591, 0.3863)$ and $(\lambda, \mu) = (0.521, 0.2768)$, respectively, but increases monotonically for $\rho = 2.4$.

Fig. S1 shows the μ^* decreasing with ρ: μ^* increases with λ for $\rho = 2.4$, whereas its minimum is in the middle ranges of λ for $\rho = 1.2$. The form of the $\mu^*(\lambda)$ function explains why the system gives longer chains under weak inhibition for $\rho = 2.4$, but middle/strong inhibition for $\rho = 1.2$ in Fig 5 and Fig 6 in the main part of the manuscript.

Marginal stability of the inactive state

The structure of the system equations makes the eigenvalues easy to compute. With $\xi = (\xi_1, ...\xi_N)$ is a vertex $(\xi_j = 0 or 1)$, the eigenvalue at ξ along the coordinate axis x_k reads:

$$\sigma_k = (-1)^{\xi_k}(-\mu\xi_k - I - \lambda \sum_{j=1}^{N} \xi_j + \sum_{j=1}^{N} J_{k,j}^{max} s_j \xi_j). \quad \text{(S8)}$$

The eigenvalue of the complete inactive state ($\xi_j = 0$ for all i) is controlled by the parameter I ($\sigma_k = -I$). Our choice of parameters, that is $I = 0$, guarantees the marginal stability of the complete inactive state.

References

1. Aguilar C, Chossat P, Krupa M, F L. Latching dynamics in neural networks with synaptic depression. PLoS One. 2017;12(8):e0183710. doi:https://doi.org/10.1371/journal.pone.0183710.
